# Quality appraisal of clinical practice guidelines for attention deficit hyperactivity disorder: a systematic review using the appraisal of guidelines for research and evaluation (AGREE II) instrument

**DOI:** 10.3389/fpsyt.2025.1576538

**Published:** 2025-06-16

**Authors:** Muhammad Dwi Wahyu, Atsunori Sugimoto, Ekachaeryanti Zain, Faisal Budisasmita Paturungi Parawansa, Hiroyuki Kasahara, Kiyohiro Yoshinaga, Jun Egawa

**Affiliations:** ^1^ Department of Psychiatry, Niigata University Graduate School of Medical and Dental Sciences, Niigata, Japan; ^2^ Department of Community Psychiatric Medicine, Niigata University Graduate School of Medical and Dental Sciences, Niigata, Japan; ^3^ Department of Psychiatry, Niigata Psychiatric Center, Nagaoka, Japan; ^4^ Department of Psychiatry, Faculty of Medicine, Mulawarman University, Samarinda, Indonesia; ^5^ Department of Psychiatry, Niigata University Medical and Dental Hospital, Niigata, Japan

**Keywords:** ADHD, guideline, systematic review, AGREE II, PRISMA

## Abstract

**Objectives:**

Attention deficit hyperactivity disorder (ADHD) can significantly impact multiple life conditions across the lifespan. Reliable clinical practice guidelines (CPGs) are crucial for the clinical decision-making for the diagnosis and management of ADHD. This study aimed to assess the quality of current CPGs for the diagnosis and management of ADHD.

**Methods:**

We conducted a systematic literature search within Pubmed, Google Scholar, the Agency for Healthcare Research and Quality, Dynamed, the National Institute for Health and Care Excellence (NICE), the National Health and Medical Research Council (NHMRC), and other local and online databases started January 19, 2022. We assessed the guideline quality using the Appraisal of Guidelines for Research and Evaluation (AGREE II) instrument. All of the included guidelines were critically appraised by five independent reviewers. We also evaluated the interrater reliability of each AGREE II domain and the overall domain score by calculating the intraclass correlation coefficient (ICC) using IBM SPSS Statistics version 28.

**Results:**

A total of 11 CPGs were included in the study. The majority of the CPGs achieved the highest score in domain 4 “Clarity of Presentation” (mean ± standard deviation, 73.73% ± 12.5%). The domains that achieved the lowest scores were domains 5 “Applicability” (mean ± standard deviation, 45.18% ± 16.4%) and 3 “Rigor of Development” (mean ± standard deviation, 51.09% ± 24.1%). The American Academy of Pediatrics (AAP), NICE, and the Malaysian Health Technology Assessment Section (MAHTAS) CPGs were identified as the strongly recommended guidelines. All AGREE II domains yielded varied interrater reliability results; the full domain ICC ranged from 0.265 (95% confidence interval, −0.470 to 0.665) to 0.758 (95% confidence interval, 0.515 to 0.889).

**Conclusions:**

Our appraisal indicated that the quality of current ADHD guidelines is varied, and three CPGs were classified as strongly recommended. Our findings offer relevant healthcare providers valuable insight into the appropriate selection of ADHD guidelines in clinical practice.

**Systematic review registration:**

https://inplasy.com/inplasy-2022-8-0001/, identifier INPLASY202280001

## Introduction

Attention deficit hyperactivity disorder (ADHD) is a neurodevelopmental disorder across the lifespan, with an estimated global prevalence of 1.6%–5% in 2024 (1, 2). ADHD is known to impair academic, occupational, and social functioning, and is associated with increased risk of accidents, higher mortality rate, and long-term economic burden if left unidentified or untreated (3–9).

Despite its high burden, underdiagnosis and undertreatment remain global challenges (10–12). These issues often arise from variations in the diagnosis and management practices due to lack of specificity in assessment tools, inconsistent research findings, methodological gaps, and lack of high-quality data comparing treatments (12, 13). These discrepancies can lead to both over- and underdiagnosis, increasing the risk of inappropriate care (12–15). Such variability underscores the importance of comprehensive, evidence-based Clinical Practice Guidelines (CPGs) that assist healthcare practitioners in making decisions about appropriate healthcare (16, 17).

While numerous ADHD CPGs have been published in the past decade, their recommendations often diverge, particularly regarding diagnostic tools, treatment thresholds, and non-pharmacological interventions (18). A previous study by Bukstein reviewed nine widely used CPGs and reported inconsistencies in recommendations across key domains, despite their complementary contents (19). In contrast, Sugimoto and Someya observed consistent approaches in diagnostic criteria and treatment integration across included guidelines, the National Institute for Health and Care Excellence (NICE), the University of Michigan Health System (UMHS), and the Canadian ADHD Resource Alliance (CADDRA) CPGs (20). However, neither study assessed the methodological rigor or transparency of these guidelines, including development processes, patient values, stakeholder involvement, or potential biases such as conflict of interest. This is concerning, as CPGs with such limitations and biases may compromise their clinical utility or applicability across diverse healthcare settings (21, 22). Therefore, reliable CPGs are essential to support consistent, evidence-based practice and improve treatment outcomes (17, 23).

To address this, it is critical to assess the quality of available CPGs using a validated appraisal tool. The Appraisal of Guidelines for Research and Evaluation (AGREE II) instrument provides a standardized framework to evaluate guideline development processes across six domains and has been widely endorsed internationally for this purpose (24–27).

Although previous studies have applied the AGREE II tool to ADHD CPGs, these reviews covered a limited number of CPGs and have not captured newly updated or developed guidelines published after 2018 (28, 29). Since then, key guidelines—such as those from NICE (30) and the American Academy of Pediatrics (AAP) (31)— have been revised in 2019, and others newly introduced. These updated documents may reflect evolving practices and thus warrant re-evaluation.

Therefore, this systematic review aims to evaluate the quality of ADHD CPGs published between 2012 and 2024 using the AGREE II instrument. By systematically evaluating the quality, recommendations, and contents of existing CPGs, this review provides practical insights to inform relevant stakeholders and guide future guideline development across diverse healthcare systems.

## Methods

This systematic review implemented Preferred Reporting Items for Systematic Reviews and Meta-Analyses (PRISMA 2020). The PRISMA 2020 checklist is available in the **Supplementary Material** (**Supplementary Table S1**) (32). The review protocol was registered in the International Platform of Registered Systematic Review and Meta-analysis Protocols (INPLASY202280001) and is available in **Supplementary File S1** (33). The title differs slightly from the registered protocol to emphasize the quality-appraisal focus, in line with peer-review recommendations.

### Eligibility criteria

This systematic review focused on published CPGs for the diagnosis and management of ADHD. CPGs were eligible for inclusion if they fulfilled the following criteria: (1) focused on the diagnosis and/or management of ADHD; (2) evidence-based CPG involving recommendations or statements; (3) the latest version; (4) full-text accessibility; (5) original sources; (6) English or English translated; (7) issued or endorsed by national or international scientific societies or government organizations; (8) published by an organization or group in a CPG database, peer-reviewed journal, or organization that comprises the relevant authorities (e.g., the ministry of health and academic organizations); (9) published between January 1, 2012 and December 31, 2021 (this period was selected according to a previous study that showed that most CPGs published before 2012 demonstrated poor compliance with Institute of Medicine standards) (34). Then, we extended our search using all of the above search methods and eligibility criteria to identify potential CPGs published between 2022 and 2024. As of 2025, no updated versions of the 11 included CPGs were identified. One new CPG was identified and developed in 2023 by the Australian ADHD Professionals Association (AADPA) (35). However, this guideline adopted the content of the existing UK NICE CPG using the ADAPTE II framework. Because our inclusion criteria required original source CPGs, we retained the National Health and Medical Research Council (NHMRC) 2012 CPG as the most recent eligible Australian CPG.

CPGs were excluded on the following criteria: fewer than three authors; a relevant publication summarizing, reporting, or reviewing the CPGs; implementing CPGs but focused only on specific or specialized ADHD problems. Informed consent was unnecessary because no humans were involved in the study. Although ethical approval was not mandatory, the Ethics Committees of Niigata University approved this study protocol (approval No. 2021-0360). We used the Population, Interventions, Professions, Outcomes, and Healthcare Settings (PIPOH framework), which is a comprehensive framework developed by the ADAPTE collaboration (36), to develop the following set of variables.

1. Population: children, adolescents, and/or adults are being assessed for an ADHD diagnosis.
2. Interventions: CPGs are focused on the diagnosis (including complaints of the parent, teacher, or adolescent, signs and symptoms, history and physical examination, psychological tools, and investigations) and/or comorbidities and management of ADHD (including pharmacological treatment, psychological and behavioral interventions, adverse effects of treatment, treatment of adverse effects, monitoring and follow-up, special cases, complementary medicine, the transition of care between child and adult, and psychosocial rehabilitation).
3. Professions: physicians (including psychiatrists, pediatricians, neurologists, medical rehabilitation specialists, general practitioners, clinical psychologists, pharmacists, nurses, dieticians, occupational therapists, and community workers) and/or the targeted population.
4. Outcomes: quality of life, ADHD symptom deterioration, functional status, peer and family relationships, academic performance, long-term side effects of stimulant medications, and/or further complications.
5. Healthcare settings: primary, secondary, and tertiary care settings.

This framework, alongside the eligibility criteria, helped us to identify relevant ADHD CPGs.

### Search strategy

The database search was started on January 19, 2022, and repeated on April 11, 2025, to capture any newly published guidelines. Three reviewers (MD, EZ, and FP) searched for CPGs in literature databases including PubMed and Google Scholar. The keywords included “attention-deficit/hyperactivity disorder,” “ADHD,” “guideline,” “practice guideline,” “clinical practice guideline,” “practice parameter,” “guidance,” and “recommendations,” as detailed in **Supplementary Tables S2**, **S3**. Then, we repeated the search to minimize the risk of missing potentially relevant studies. Additionally, we checked the references of the guidelines (snowball technique) via reference tracking and citation searching to identify any additional potential guidelines. Further, we explored guideline databases from national and international scientific societies and government organizations, including Evidence-Based Medicine Clinical Outcomes DynaMed Plus, the American Agency for Healthcare Research and Quality, National Guideline Clearinghouse, Guidelines International Network, Scottish Intercollegiate Guidelines Network, NICE, APA, NHMRC, European Psychiatric Association (EPA), and various Ministries of Health websites (see **Supplementary Table S4**). To ensure thoroughness, we also browsed local and national websites to identify any other relevant CPGs that might not have been captured in these databases.

### Study selection process

**Figure 1:**
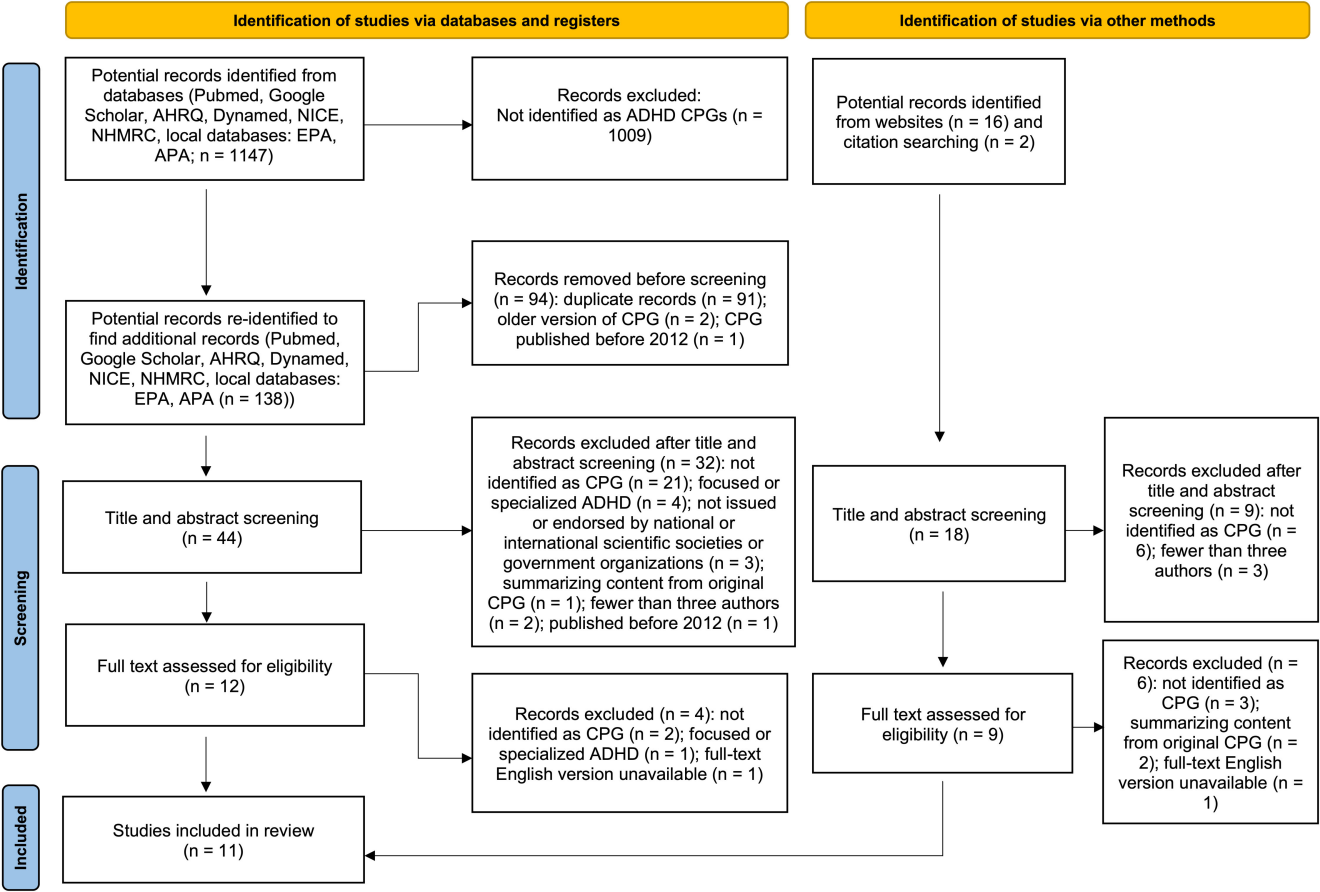
PRISMA 2020 flow diagram.

Three reviewers independently screened the title and abstract of each potential record identified from the databases search using the inclusion and exclusion criteria. Any disagreements were settled through discussion with a fourth reviewer (AS). Full-text documents were obtained for studies that met the criteria or required further assessment beyond the title and abstract. The three independent reviewers screened the full-text documents to identify eligible CPGs. Any disagreement was resolved through discussion with all authors. Characteristics of guidelines that were excluded during this process were duplicate records, previous editions, and those published before 2012 (these are detailed in **Figure 1**).

### Data extraction

The three reviewers extracted the data from all eligible CPGs. Data extraction comprised the guideline title, developer organization, year of publication, country of publication, retrieval source, URL or DOI, comments (if applicable), and other relevant guideline information. We also extracted the AGREE II domain scores and two overall assessments from each guideline appraisal.

### Guidelines quality assessment

The quality assessment of the CPGs was conducted online using the AGREE II instrument, My AGREE PLUS (https://www.agreetrust.org/resource-centre/agree-plus/). AGREE II is a widely used and validated tool that assesses the methodological rigor and transparency of the development of a guideline. It comprises 23 items organized into six quality domains: Scope and Purpose (items 1–3), Stakeholder Involvement (items 4–6), Rigor of Development (items 7–14), Clarity of Presentation (items 15–17), Applicability (items 18–21), and Editorial Independence (items 22–23). These 23 items target various aspects of CPG quality. Each item was rated on a 7-point scale, from 1 (strongly disagree) to 7 (strongly agree). The maximum possible score is 7, which indicates that the quality of reporting is exceptional and the CPG fulfills all criteria and considerations. The minimum possible score is 1, which indicates that no information is relevant to the AGREE II item. A score of 2–6 suggested that the reporting of the AGREE II item did not meet all criteria or considerations and was dependent on the amount of information provided by the CPGs for that item. The AGREE II also includes two final overall assessment items that require the appraiser to make an overall judgment of the CPGs based on the ratings of the 23 items.

### Rating of AGREE II domains

Five AGREE II assessors were chosen: a child and adolescent psychiatrist (AS), a general psychiatrist (EZ), a psychiatric resident (HK), a neurologist (FP), and a medical doctor (MD). The assessors used the AGREE II handbook and the audio-visual training to thoroughly familiarize themselves with the methodology before assessing the eligible CPGs (24, 37). The AGREE-II checklist was used to assess each CPG (38). Each assessor independently scored all eligible CPGs and their Supplementary Files and links to web pages relating to the methodology or implementation of the guidelines.

### Data synthesis and analyses

Descriptive statistics were used to summarize the CPG characteristics, the clinical content regarding the diagnosis and management of ADHD, and the AGREE II instrument assessment results. The summary measures (i.e., mean, median, and standard deviation) and Shapiro-Wilk *p*-value were calculated for each AGREE II domain for each CPG.

The total domain scores were scaled as a percentage of the maximum possible score for each domain using the following formula: (obtained score − minimum possible score)/(maximum possible score − minimum possible score) × 100 = percentage (range from 0% to 100%). There is currently no consensus on the threshold for AGREE II instrument domain scores for high quality (24). Previous studies have considered domain 3 (rigor of development) as the most important domain of the AGREE II instrument (39–43). However, there is considerable variability in the cut-off for determining the quality of CPG when using the AGREE II instrument; moreover, there is no empirical evidence linking quality ratings to specific implementation results (24). Nevertheless, previous studies have used a cut-off of 60% of AGREE II domain scores (40, 41, 44). Therefore, we formulated three categories by implementing a 60% cut-off and using domain 3 as a requisite to classify the quality of CPGs: “strongly recommended CPGs (++)” were defined as those that obtained > 60% for at least four domains (including domain 3) and ≥ 30% for the remaining domains; “not recommended CPGs (–)” if four or more domains were < 30% or domain 3 was < 30%; all other cases were defined as “recommended CPGs (+).”

The ratings of all assessors were measured using the intraclass correlation coefficient (ICC), a reliability measurement that has been widely used to evaluate test-retest, intrarater, and interrater reliability. To assess interrater reliability, we used a two-way mixed effects model with rater average per domain and overall rating consistency to calculate an average ICC for every three sets of raters for each CPG. The null hypothesis was that the ICC would equal 0. Therefore, if the values were more than 0.5 (*p* ≥ 0.05), the ICC differed significantly from 0. We interpreted the ICC as follows: an ICC < 0.4 implied poor reliability, an ICC between 0.4 and 0.75 implied moderate reliability, and an ICC ≥ 0.75 indicated excellent reliability (45–49). All statistical analyses were performed using IBM SPSS Statistics version 28.

## Results

### Included ADHD CPGs

We obtained 1147 records from the literature search of the systematic review databases (**Figure 1**). Further websites and citation searching identified 18 potential records (total n = 1165). After removing duplicates and re-identifying additional potential records, 62 titles and abstracts were screened, and 21 full-text records were assessed for eligibility. Subsequently, 10 studies were excluded, which resulted in the inclusion of 11 CPG documents developed by the AAP (31), Singapore Ministry of Health (SMOH) (50), CADDRA (51), NICE (30), NHMRC (52), the Malaysian Health Technology Assessment Section (MAHTAS) (53), UMHS (54), the Institute for Clinical Systems Improvement (ICSI) (55), the Indian Academy of Pediatrics (56), the British Association for Psychopharmacology (BAP) (57), and EPA (12) (**Table 1**). In addition, we also provide detailed information on excluded guidelines and the reasons is available in **Supplementary Table S5**.

**Table 1:** General characteristics of ADHD CPGs.

No	Affiliation and Title	Year of Publication	Country of Origin	Level of Development	Guideline Development Group	Target Population	Number of References	Funding
1	AAP (Clinical Practice Guideline for the Diagnosis, Evaluation, and Treatment of Attention-Deficit/Hyperactivity Disorder in Children and Adolescents)	2019	USA	National	Specialty society	Children, and adolescents	373	–
2	SMOH (Academy of Medicine-Ministry of Health Clinical Practice Guidelines: Attention Deficit Hyperactivity Disorder)	2014	Singapore	National	Government	Children and adolescents	250	–
3	CADDRA (Canadian ADHD Practice Guidelines 4.1 Edition)	2020	Canada	National	Independent, not-for-profit association	Children, adolescents, and adults	496	CADDRA
4	NICE (Attention Deficit Hyperactivity Disorder: Diagnosis and Management)	2018 (updated in 2019)	UK	National	Government	Children, adolescents, and adults	2941	NICE
5	NHMRC (Clinical Practice Points on the Diagnosis, Assessment and Management of Attention Deficit Hyperactivity Disorder in Children and Adolescents)	2012	Australia	National	Government	Children and adolescents	112	NHMRC
6	MAHTAS (Clinical Practice Guidelines Management of Attention-Deficit/Hyperactivity Disorder in Children and Adolescents (Second Edition))	2020	Malaysia	National	Government	Children, adolescents, and adults	77	Ministry of Health Malaysia
7	UMHS (Guidelines for Clinical Care Ambulatory Attention-Deficit Hyperactivity Disorder)	2013 (updated in 2019)	USA	Local	Expert group made up of members of the university	Children, adolescents, and adults	16	–
8	ICSI (Health Care Guideline Diagnosis and Management of Attention Deficit Hyperactivity Disorder in Primary Care for School-Age Children and Adolescents)	2012	USA	Local	Independent, not-for-profit association	Children and adolescents	123	ICSI
9	IAP (Consensus Statement of the Indian Academy of Pediatrics on Evaluation and Management of Attention Deficit Hyperactivity Disorder)	2017	India	National	Specialty society	Children and adolescents	30	–
10	BAP (Evidence-based Guidelines for the pharmacological management of attention deficit hyperactivity disorder: Update on recommendations from the British Association for Psychopharmacology)	2014	UK	National	Specialty society	Children, adolescents, and adults	176	Janssen, Lilly, and Flynn-Pharma
11	EPA (Updated European Consensus Statement on Diagnosis and Treatment of Adult ADHD)	2019	European Countries	International	Specialty society	Adult	353	–

ADHD, Attention deficit hyperactivity disorder; AAP, American Academy of Pediatrics; SMOH, the Singapore Ministry of Health; CADDRA, Canadian ADHD Resource Alliance; NICE, National Institute of Health and Care Excellence; NHMRC, National Health Medical Research Center; MAHTAS, Malaysian Health Technology Assessment Section; UMHS, University of Michigan Health System; ICSI, Institute of Clinical System Improvement; IAP, Indian Academy of Pediatrics; BAP, British Association for Psychopharmacology; EPA, European Psychiatric Association; UK, United Kingdom; USA, United States of America; CPG, clinical practice guidelines.

### CPG characteristics

**Table 1** describes the characteristics of the included CPGs. All CPGs were published from 2012 to 2020. Three (30.8%) were developed in the United States of America, two (15.4%) in the United Kingdom, and the remaining were developed in Singapore, Canada, Australia, Malaysia, India, and a combination of multiple European countries. Eight (72.7%) guidelines were national development-based guidelines, two (18.2%) were local development-based guidelines, and one (9.1%) was an international development-based guideline. Four (36.4%) guidelines were developed by the country’s government, and four (36.4%) guidelines were developed by medical specialty societies or associations. Most of the included guidelines targeted children and adolescent ADHD groups (45.5%).

### Review of CPG recommendation

#### Section 1: Diagnosis of ADHD

Details of diagnostic recommendations across the included CPGs are summarized in **Table 2**. Most CPGs emphasize a comprehensive clinical interview involving multiple informants (e.g., parents, teachers, partners, or other informants) and using either the Diagnostic and Statistical Manual (DSM) of Mental Disorders and/or the International Classification of Diseases (ICD) criteria (12, 30, 31, 50–57). An exception is the BAP guideline, as diagnosis was beyond its scope. While core recommendations are generally consistent, variations exist in the specificity and consideration of guidance. For instance, some CPGs (APA, EPA, UMHS) provide detailed age-specific considerations. APA highlights diagnostic challenges in adolescents compared to young children due to less overt behavior (31), EPA emphasizes collateral information for adults with recall limitations (12). EPA and UMHS include thorough discussions on adult ADHD, suggesting retrospective symptom assessment and comorbidities (12, 54). Several CPGs (e.g., EPA, MAHTAS, NHMRC, and NICE) highlight the heterogeneity of ADHD manifestation across lifespan and challenges in consistent application of criteria, particularly under ICD versus DSM criteria (12, 30, 52, 53). These differences may contribute to inconsistencies in ADHD diagnosis across clinical settings.

**Table 2:** Recommendations of reviewed CPGs for the diagnosis of ADHD.

Assessment	AAP	SMOH	CADDRA	NICE	NHMRC	MAHTAS	UHMS	ICSI	IAP	BAP	EPA
Screening for ADHD in individuals with academic or behavioral problems and symptoms of inattention, hyperactivity, or impulsivity	**	**	**	**	**	**	**	**	*	–	**
DSM	**	*	**	**	**	**	**	**	*	–	**
ICD	–	*	–	**	**	**	–	–	–	–	*
Rating scale	*	*	**	**	–	*	*	*	*	–	*
Screening for coexisting conditions	**	**	**	*	**	*	**	**	*	–	*

**definitely recommended; *mentioned, which may be useful; -not mentioned.

ADHD, attention deficit hyperactivity disorder; AAP, American Academy of Pediatrics; SMOH, Singapore Ministry of Health; CADDRA, Canadian ADHD Resource Alliance; NICE, National Institute of Health and Care Excellence; NHMRC, National Health Medical Research Center; MAHTAS, Malaysian Health Technology Assessment Section; UMHS, University of Michigan Health System; ICSI, Institute of Clinical System Improvement; IAP, Indian Academy of Pediatrics; BAP, British Association for Psychopharmacology; EPA, European Psychiatric Association; DSM, Diagnostic and Statistical Manual of Mental Disorders; ICD, International Statistical Classification of Diseases and Related Health Problems.

Most CPGs do not include rating scales within their formal diagnostic. However, several tools—such as the ADHD Self-report Scale, the Conners Rating Scale, the Child Behavior Checklist, the Vanderbilt Assessment Scales, and the Strengths and Difficulties Questionnaire—are frequently mentioned as optional aids for assessing ADHD symptoms (12, 30, 31, 50–55, 57). The use of these scales varies across CPGs and likely reflect cultural and systemic differences in how ADHD diagnosis is approached and implemented in practice.

Regarding physical and additional investigations, most CPGs do not endorse routine use unless clinically indicated. Some guidelines mention physical examinations, such as checking vital signs, height, weight, and vision/hearing examination (53–56), while others do not specify examination type (31, 51). These assessments are typically suggested in the context of diagnosis or differential diagnosis, especially when symptoms may mimic ADHD. For neurophysiological, laboratory, and imaging investigations, all CPGs acknowledge variability but generally discourage their routine because of insufficient and/or contradictory evidence. For example, most CPGs described that laboratory and other investigations cannot be recommended because of insufficient evidence (12, 30, 51, 52, 55, 57). Similarly, electroencephalography and magnetic resonance imaging are not routinely indicated (53). However, some CPGs suggest thyroid hormone function tests if indicated (31, 50). These suggestions aim to avoid unnecessary testing, promoting cost-effective use of healthcare resources. However, the lack of recommended objective diagnostic tools may increase the risk of misdiagnosis, particularly when ADHD symptoms overlap with other psychiatric or medical conditions. Given the reliance on subjective clinical judgment, future efforts should focus on identifying reliable biomarkers or objective measurements to improve diagnostic accuracy and standardization.

#### Section 2: Management of ADHD

All CPGs categorize management recommendations into two main categories: pharmacological and non-pharmacological treatments. In addition, all CPGs categorize recommendations according to age group: preschool-aged children (approximately 4–6 years old), middle school-aged children (approximately 6–12 years old), adolescents (approximately 12–18 years old), and adults (over 18 years old).

### Preschool-aged children

Several CPGs recommend non-pharmacological treatment before starting any pharmacological treatment, such as parent training and/or classroom intervention (30, 31, 50, 56). If preschool-aged children with ADHD require pharmacological treatment because non-pharmacological treatments are ineffective, several CPGs recommend stimulants, such as methylphenidate (MPH) short or long-acting release as the drug of choice (31, 50, 56), or referral to a tertiary healthcare service that specializes in managing ADHD for further analysis (30, 52, 53).

### Middle school-aged children

For middle school-aged children with ADHD, most CPGs recommend stimulants as first-line pharmacological treatment, such as MPH, amphetamine, and lisdexamphetamine (30, 31, 50–53, 55–57). Several CPGs recommend the specific drug type, such as short or immediate release (30, 50), modified release (30), extended release (50), and long-acting release (51), whereas others do not offer a specific drug type in their recommendation (31, 52–55). Most CPGs recommend atomoxetine, a non-stimulant drug, as a second-line drug treatment (30, 31, 50, 51, 53, 54, 56, 57). Moreover, several CPGs suggest guanfacine (30, 31, 51, 54), bupropion (54), risperidone (54), or clonidine (31, 54) as adjunctive and/or alternative second-line treatment options. Most CPGs recommend that the clinician encourage non-pharmacological treatment alongside pharmacological treatment as combination or multimodal management, although non-pharmacological treatment alone without medication is not recommended (30, 31, 50–57). Non-pharmacological interventions may include parent training and/or school-based interventions (31, 50, 53–57), educating parents, carers, and/or teachers about ADHD (30, 50, 51, 54, 57), additional group-based support for parents, carers, and/or teachers (30, 50), cognitive behavioral therapy (30, 50, 53), occupational therapy (53), or social skills training (50, 53, 55). An adjunctive care plan, such as educational interventions, individualized instructional support for patients, and/or adjustment between different settings (e.g., home, school, and work) and relationship-based care for young people, is recommended by several CPGs for this age group (31, 51, 52, 54).

### Adolescents

For adolescents with ADHD, most CPGs recommend the same drug treatments as those recommended for middle school-aged children. However, recommendations for non-pharmacological management differ from those for middle school-aged children. For example, several CPGs recommend evidence-based training interventions and/or behavioral therapy (31), or cognitive behavioral therapy alongside medication (52), whereas other CPGs recommend the same non-pharmacological management options as those recommended for middle school-aged children (30, 50, 51, 53, 55–57). Several CPGs also recommend that clinicians plan the transition phase from young person to adult in advance alongside discussions with the patient and their family to ensure the continuation of ADHD treatment throughout the lifespan (12, 30, 50, 52).

### Adults

For adult cases, most CPGs recommend psychoeducation (12, 51) or environmental modification (30) as first-line management before considering medication. If medication is needed because of persistent symptoms causing significant problems, stimulants are recommended as the first-line drug treatment, which include amphetamine, lisdexamphetamine, and MPH (12, 30, 51, 57). Although not all CPGs specify drug type, several CPGs recommend long-lasting or extended-release formulations (12, 51). For second-line drug treatment for adults with ADHD, several CPGs recommend atomoxetine (12, 30, 51, 57). If both first- and second-line treatments remain ineffective, several CPGs recommend guanfacine, clonidine, bupropion, tricyclic antidepressants, reboxetine, atypical antipsychotics, or other medications not considered first- or second-line treatments. However, it is recommended that advice is sought from tertiary healthcare services that specialize in managing ADHD (30). One CPG states that evidence for these medications remains limited (12). Cognitive behavioral therapy is recommended as an adjunctive treatment for combination or multimodal management alongside medication, but not as the sole treatment for adults with ADHD (12, 30).

### Baseline assessment and monitoring

Several CPGs recommend baseline assessments, such as cardiovascular examination (blood pressure and heart rate), height, weight, and/or body mass index, either before or during the monitoring phase of pharmacological treatment, especially stimulants (30, 31, 50–53, 55). In addition, some CPGs recommend careful drug titration (30, 31, 50–52, 57). Furthermore, periods off medication, as referred to by some CPGs as a “drug holiday,” are also recommended to evaluate the benefits and risks of continuing treatment (30, 50, 52, 57).

### Dietary intervention

Several CPGs described dietary intervention for ADHD, although these approaches lack evidence, require further research, or may only be used as adjunctive treatments (30, 50, 53, 57). For example, some CPGs did not include dietary interventions, such as supporting additive and sugar elimination, in their recommendation because of insufficient evidence (50, 51, 53), whereas some CPGs explicitly stated that restrictive elimination diets (e.g., artificial colors and additives) are not recommended or advisable (30, 50).

Contradictive recommendations were also found for fatty acid supplementation; one CPG did not recommend it (30, 50), whereas another recommended it as an adjunctive treatment (30, 50). Furthermore, referral to a dietitian may be considered as additional management (30, 50).

### Supplementary intervention

Several CPGs recommend other supplementary therapies for ADHD, although such approaches lack evidence, require further investigation, or may only be used as adjunctive treatments (30, 50, 53, 57). For example, several CPGs are uncertain about or do not recommend the use of neurofeedback and computer-assisted cognitive training because of a lack of evidence (50, 51, 53). However, other CPGs suggest the use of neurofeedback and referral to an occupational therapist or pediatrician as an adjunctive treatment that could benefit the parent/carer and child or young person with ADHD (30, 50). Detailed descriptions of the non-pharmacological and pharmacological management recommendations of the CPGs are provided in **Tables 3** and **4**.

**Table 3:** Recommendations of the reviewed CPGs for the non-pharmacological management of ADHD.

Intervention	AAP	SMOH	CADDRA	NICE	NHMRC	MAHTAS	UHMS	ICSI	IAP	BAP			EPA
For subjects													
Classroom- or school-based intervention/individualized instructional support	**	**	**	**	**	**	**	**	*		–	–	
Group-based support	–	–	–	**	–	–	*	**	–		–	–	
Psychoeducation	–	**	**	**	–	–	*	**	–		–	**	
Behavioral training	**	–	**	–	*	**	**	**	*		–	–	
Occupational therapy	–	*	*	–	–	**	–	–	*		–	–	
Social skills training	–	*	*	–	–	**	*	**	–		–	–	
CBT	–	*	*	*	*	**	*	**	–		–	*	
Cognitive remediation	–	*	–	–	–	–	–	–	–		–	–	
Mindfulness	–	–	*	–	–	–	–	–	–		–	–	
Environmental modification	–	–	**	**	–	–	–	–	–		–	–	
Neurofeedback	–	*	–	–	–	–	–	–	–		–	–	
Fatty acid or Omega-3	–	*	–	x	–	–	*	–	–		–	–	
Restrictive elimination diet or dietary modification	–	x	–	x	–	–	*	–	–		–	–	
For parents, carers, teachers, and/or other significant others													
Psychoeducation	–	**	**	**	–	–	*	**	–	**			–
Parent training	**	**	*	**	*	**	*	**	–	–			–
Family therapy	–	**	*	–	*	–	*	*	–	–			–
Group-based support	–	–	–	**	–	–	*	**	–	–			–

**definitely recommended; *recommended as additional consideration and/or not as a sole treatment; x not recommended and/or not advisable; -not mentioned.

ADHD, attention deficit hyperactivity disorder; AAP, American Academy of Pediatrics; SMOH, Singapore Ministry of Health; CADDRA, Canadian ADHD Resource Alliance; NICE, National Institute of Health and Care Excellence; NHMRC, National Health Medical Research Center; MAHTAS, Malaysian Health Technology Assessment Section; UMHS, University of Michigan Health System; ICSI, Institute of Clinical System Improvement; IAP, Indian Academy of Pediatrics; BAP, British Association for Psychopharmacology; EPA, European Psychiatric Association; CBT, cognitive behavioral therapy.

Note that each guideline may recommend referring to a tertiary healthcare center or specialized healthcare (e.g., dietitian or occupational therapist) to assess and provide further appropriate non-pharmacological management; moreover, recommendations depend on the condition of each patient and the preferences of the parents, carers, teachers, and/or significant others.

**Table 4:** Recommendations of the reviewed CPGs on the pharmacological management of ADHD.

Formulation Agents or Active Ingredients	AAP	SMOH	CADDRA	NICE	NHMRC	MAHTAS	UHMS	ICSI	IAP	BAP	EPA
Short-acting or immediate-release MPH	1st for preschool children and children	1st for children and adolescents	2nd or augment for children, adolescents, and adults	1st for children, adolescent, and adult	1st for children and adolescents/TAS for preschool children	1st for children and adolescents/TAS for preschool children	1st for children and adolescents/TAS for preschool children	1st for children and adolescents	1st for children and adolescents	1st in children and adults	1st for adults
Long-acting, modified-release, or extended-release MPH	1st for preschool children and children	1st for children and adolescents	1st for children, adolescents, and adults	1st for children, adolescent, and adult	1st for children and adolescents/TAS for preschool children	1st in children and adolescents/TAS for preschool children	1st for children and adolescents/TAS for preschool children	1st for children and adolescents	1st for children and adolescents	1st for children and adults	1st for adults
Dexmethylphenidate	1st for preschool children and children	–	–	–	–	–	1st for children and adolescents	1st for children and adolescents	–	–	1st for adults
Amphetamine	1st for children	–	–	–	–	–		–	2nd for children and adolescents	–	–
Mixed amphetamine salts	1st for children	–	1st for children, adolescents, and adults	–	–	–	1st for children and adolescents	1st for children and adolescents	–	–	–
Amphetamine sulphate	1st for children	–	–	–	–	–	1st for children and adolescents	–	–	–	–
Dextroamphetamine	1st for children	–	2nd or augment for children, adolescents, and adults	2nd for children, adolescents, and adults	1st for children and adolescents/TAS for preschool children	–	1st for children and adolescents	1st for children and adolescents	–	1st for children and adults	1st for adults
Lisdexamfetamine	1st for children	–	1st for children, adolescents, and adults	2nd for children and adolescents/1st for adults	–	–	1st for children and adolescents	1st for children and adolescents	–	1st for children and adults	1st for adults
Modafinil	–	–	–		–	–	–	–	–	3rd for adults	–
Atomoxetine	1st for children	2nd for children and adolescents	2nd or augment for children, adolescents, and adults	3rd for children, adolescents, and adults	–	2nd for children and adolescents	2nd for children and adolescents	1st for children and adolescents	2nd for children and adolescents	2nd for children and adults (1st if any contraindications)	2nd for adults
Bupropion	–	Augment for children and adolescents	–	–	–	–	2nd or augment for children and adolescents	TAS for children and adolescents	–	3rd for adults/TAS for children and adolescents	–
Trazodone	–	–	–	–	–	–	–	–	–	–	–
Tricyclic antidepressant	–	Augment for children and adolescents	–	–	–	–	–	TAS for children and adolescents	–	3rd for adults	–
Imipramine	–	Augment for children and adolescents	–	–	–	–	–	–	–	–	–
Desipramine	–	–	–	–	–	–	–	–	–	–	–
Clonidine	1st for children	–	–	TAS for children	–	–	2nd for children and adolescents	1st/TAS for children and adolescents	–	3rd for adults/TAS for children and adolescents	–
Guanfacine	1st for children	–	2nd or augment for children and adolescents	3rd for adolescents/TAS for adults	–	–	2nd for children and adolescents	1st/TAS for children and adolescents	–	3rd for adults	–
Atypical antipsychotic	–	–	–	TAS (unspecified)	–	–	–	–	–	–	–
Risperidone	–	–	–	–	–	–	–	–	–	–	–
Aripiprazole	–	–	–	–	–	–	–	–	–	–	–
Carbamazepine	–	–	–	–	–	–	–	–	–	–	–

1st: definitely recommended as first-line treatment and/or main drug of choice; 2nd: definitely recommended as second-line treatment; 3rd: definitely recommended as third-line treatment or alternative drug of choice after first- and second-line treatments; augment: mentioned as an adjunctive drug of choice or additional alternative drug of choice outside of first-, second- and third-line drugs of choice; -: not mentioned in the recommendation.

ADHD, attention deficit hyperactivity disorder; AAP, American Academy of Pediatrics; SMOH, Singapore Ministry of Health; CADDRA, Canadian ADHD Resource Alliance; NICE, National Institute of Health and Care Excellence; NHMRC, National Health Medical Research Center; MAHTAS, Malaysian Health Technology Assessment Section; UMHS, University of Michigan Health System; ICSI, Institute of Clinical System Improvement; IAP, Indian Academy of Pediatrics; BAP, British Association for Psychopharmacology; EPA, European Psychiatric Association; MPH, methylphenidate; TAS, considered by tertiary ADHD service.

Preschool children: preschool-aged children aged 4–6 years; children: middle school-aged children aged 6–12 years; adolescents: young adults aged 12–18 years; adults: aged 18 years or older; unspecified: unspecified age group.

Note that each guideline may recommend secondary, tertiary, or other levels of treatment, even for off-label prescriptions in their country. Drug indication for ADHD patients should be checked for each country. Some guidelines do not explicitly mention the type of MPH recommended; such cases are listed as MPH alone. Dextroamphetamine = Dexamphetamine.

### Quality appraisal based on AGREE II domains

**Figure 2:**
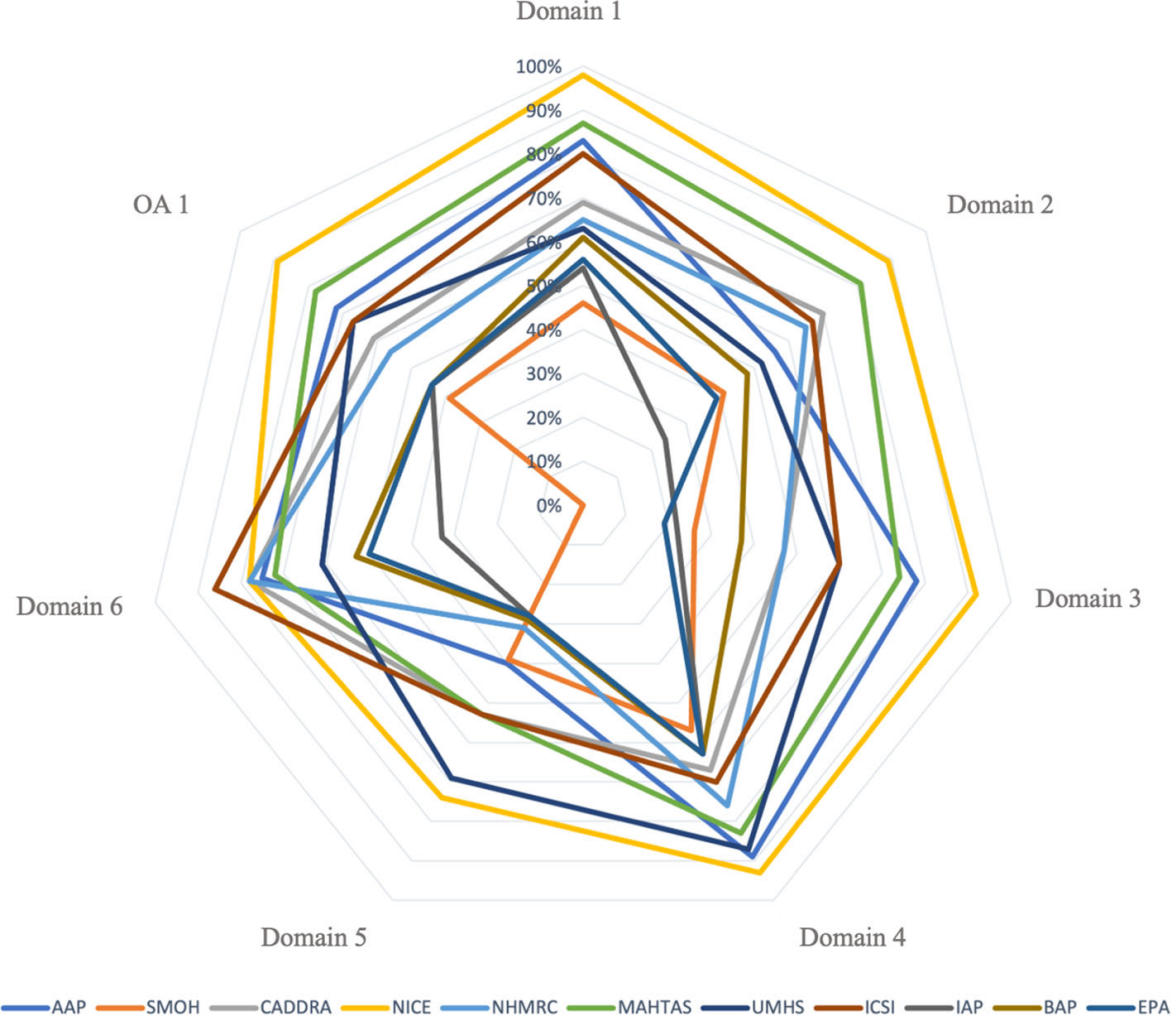
AGREE II domain scores for Included ADHD CPGs.

The scores of each AGREE II domain, overall assessments, and summary measures (mean, median, and standard deviation) are provided in **Supplementary Table S6**. The domains that reached the highest and lowest average score across all 11 CPGs were domain 4 “Clarity of Presentation” (73.73%) and domain 5 “Applicability” (45.18%), respectively. The average score for Domain 3 “Rigor of Development” ranged from 19% to 92% with a mean score of 51.09%. For overall assessment 1, the NICE CPG achieved the highest score (89%) (**Figure 2**). In addition, it was the only CPG that all three reviewers “recommended the guideline for use” without modification on overall assessment 2. Furthermore, the Shapiro-Wilk test indicated that Domain 6 “Editorial Independence” differed significantly among the 11 CPGs (*p* = 0.034).

### Quality of each CPG

To determine the quality of each CPG, we used the 60% cut-off, as detailed in **Supplementary Table S6**. Of the 11 eligible CPGs, three CPGs (the AAP, NICE, and MAHTAS CPGs) met the criteria for **“strongly recommended,”** five CPGs (the CADDRA, NHMRC, UMHS, ICSI, and BAP CPGs) were categorized as **“recommended,”** and three CPGs (the SMOH, Indian Academy of Pediatrics, and EPA CPGs) were considered **“not recommended.”**

### Interrater reliability

**Figure 3:**
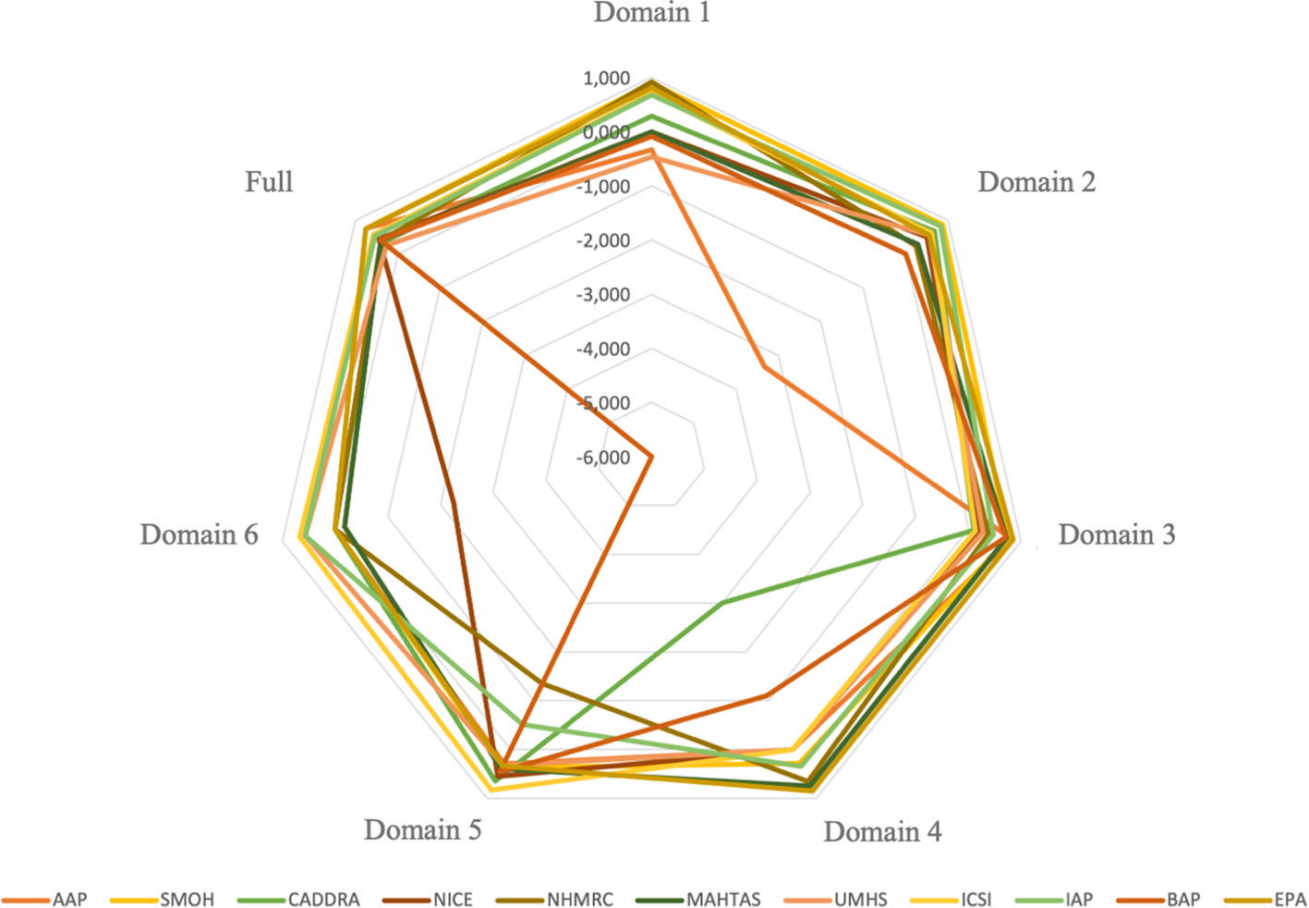
Interrater agreement of AGREE II domain scores.

The score of interrater reliability among the three raters is presented in **Figure 3**. The level of consistency among raters across individual domains illustrated varied findings. Some domain’s scores showed moderate (ICC = 0.458–0.728) to excellent consistency (ICC = 0.750–0.919). In contrast, several domains yielded 0 score, either due to zero agreement (e.g., domain 2 of the BAP, domain 6 of the AAP, and domain 6 of the EPA), consistent agreement with lack of variability (e.g., domain 1 of the MAHTAS, domains 1 and 4 of the NICE, domain 4 of the AAP, domain 4 of the UMHS, domain 4 of the ICSI, domain 6 of the SMOH, domain 6 of the CADDRA, and domain 6 of the NHMRC) or identical agreement with no variability (e.g., domain 6 of the SMOH). Notably, some domains yielded negative ICC values (range: –0.083 to –6.000), indicating greater variance within intraraters than between interraters. Despite the variability observed across individual domain, the overall interrater reliability, based on combined scores from all 23 AGREE items, ranged from moderate to excellent interrater reliability (ICC = 0.410–0.758), as detailed in **Supplementary Table S7**.

## Discussion

This systematic review evaluated 11 CPGs for ADHD using the AGREE II instrument, revealing substantial variability in scores across the six AGREE II domains (**Figure 2** **;** **Supplementary Table S6**). Notably, only one CPG met the preset 60% threshold in all domains, highlighting a clear need for improvement in future ADHD CPG development. Most CPGs applied structured evidence-grading systems, with a few specifically using the Grading of Recommendations Assessment, Development, and Evaluation approach (e.g., NICE, ICSI), or similar evidence hierarchies (e.g., MAHTAS, UMHS, SMOH, AAP, BAP). On the other hand, several CPGs (IAP, NHMRC, CADDRA, EPA) did not explicitly specify their grading systems, resulting in variability in the clarity and specificity of recommendations.

### Comparisons of CPG recommendation

#### Section 1: Diagnosis of ADHD

Overall, most of the included CPGs recommend using DSM criteria more frequently than ICD for ADHD diagnosis and recommend screening individuals with academic or behavioral difficulties. However, recommendations on rating scales vary widely—only CADDRA and NICE explicitly endorse their use (30, 51), while others consider them helpful but insufficient for standalone diagnosis (see **Table 2**) (12, 31, 50, 53–56). This variability may stem from a high variety of rating scale types and the need for more research to confirm their usefulness.

#### Section 2: Management of ADHD

##### Non-pharmacological

In general, recommendations regarding the non-pharmacological management of ADHD were varied across CPGs. Psychoeducation and parent training were consistently endorsed across guidelines (30, 31, 50–55, 57). However, there were discrepancies regarding interventions such as group-based support, cognitive behavioral therapy (CBT), occupational therapy, fatty acid supplementation, dietary modification, and neurofeedback, reflecting differences in target age groups, guideline scope, and evidence appraisal (see detailed comparisons in **Table 3**).

##### Pharmacological

Pharmacological treatment recommendations across CPGs consistently endorse stimulants as first-line treatments, particularly MPH, across age groups (12, 30, 31, 50–57). Atomoxetine, a non-stimulant, is commonly recommended as a second-line treatment, though typically not for preschool-aged children (12, 50, 51, 53, 54, 56, 57). However, high variability existed in recommendations for other medications and how CPGs specify the order of recommended medications. Thus, clinicians must carefully consider age-specific recommendations and the need to stay informed about the nuances of each guideline to optimize ADHD management when choosing pharmacological treatments (see detailed comparisons in **Table 4**).

### Comparisons of each AGREE II domain

Most CPGs achieved high scores for domains 1 (scope and purpose) and 4 (clarity of presentation). For the reporting criteria for domain 1, 8 out of 11 CPGs (i.e., the AAP, CADDRA, NICE, NHMRC, MAHTAS, UMHS, ICSI, and BAP CPGs) thoroughly addressed the objective, clinical question, and target population (24). However, we found that three CPGs did not effectively target this domain owing to insufficient information on the health question and/or the target population (12, 50, 56). Of the 11 CPGs, 10 fulfilled the clarity of presentation domain criteria, which indicated that most recommendations are specific, unambiguous, and clear in their presentation of the management options for ADHD (24). The CPGs that scored poorly in this domain had poor accessibility to the key recommendations.

Our review revealed that most CPGs scored poorly for domains 3 (rigor of development) and 5 (applicability). Most CPGs failed to effectively target domain 3 because they lacked information on the inclusion and exclusion criteria used, the method used to develop the recommendations, and which method will be used to update the CPG. However, the AAP, NICE, and MAHTAS CPGs exceeded the 60% cut-off score for domain 3. These CPGs adequately detailed the search strategy methods used, evidence selection criteria, strengths and limitations of the evidence, consideration of harm and benefit to formulate recommendations, and the link between the recommendations and evidence. Furthermore, the NICE CPG development group conducted a systematic review for each clinical question. Most CPGs received a low score for domain 5 because they provided inadequate information on implementation barriers and facilitators and potential resource implications, such as the types of costs associated with implementing the recommendations. However, the NICE and UMHS CPGs received a high score for domain 5 because they included a description of the additional materials needed to implement the recommendations in practice and potential resource implications, such as cost-effectiveness. The average AGREE II score for domain 2 (stakeholder involvement) was low across all CPGs. However, five CPGs (i.e., the CADDRA, NICE, NHMRC, MAHTAS, and ICSI CPGs) adequately targeted (≥ 60%) this domain. Specifically, these five CPGs provided information about the development group members or intended users of the CPG. Six CPGs obtained a score of < 60% because they had insufficient information on the approach used to gather the views and preferences of the target population (24). We found that the majority of ADHD CPGs provided limited information on the methods used to gather the views and preferences of the target population and how outcomes affected the development and formulation of the recommendations.

The domain 6 (editorial independence) mean score was relatively high across all CPGs, and seven CPGs (i.e., the AAP, CADDRA, NICE, NHMRC, MAHTAS, UMHS, and ICSI CPGs) exceeded the 60% cut-off for this domain. These CPGs provided sufficient description regarding whether funding bodies influenced the development of the CPG and any competing interests of the developers. The four CPGs that ineffectively targeted (i.e., scored < 60%) this domain failed to mention conflicts of interest and the influence of funding bodies on the CPG development process (44).

The NICE CPG was the only CPG that adequately described all six domains according to the 60% cut-off score of the AGREE II instrument. Indeed, recent systematic reviews have also found that the NICE CPG satisfies each AGREE II instrument domain as well as the first version of the AGREE tool (28, 58, 59). Specifically, the NICE CPG was developed using internationally recognized CPG standards, such as the AGREE II criteria and the CPG Implementability Appraisal tool; moreover, the committee applied primary methodological research and evaluation conducted by NICE to the CPG. The NICE CPG comprises a search strategy, selection criteria for evidence, critical evaluation of clinical and economic evidence, a consultation and validation process, implementation concerns and resources, and transparent and clear health questions (60). The MAHTAS CPG also fulfilled all domains exceeding ≥ 70%, except for domain 5 (applicability). The developers of the MAHTAS CPG considered the AGREE II criteria when developing the CPG (53).

### Strengths and limitations of the study

The strengths of our study are our systematic search strategy to minimize the risk of missing CPGs and ensure a comprehensive review of current guidelines published between 2012 and 2024. Additionally, our study includes CPGs developed in diverse regions, such as the United States, Europe, Australia, and Asia, by internationally recognized organizations. Another strength is our use of the AGREE II checklist as a guide for the evaluation of CPG quality and assessment of methodological rigor quality, including stakeholder involvement as one of its key domains. Through this, we evaluated the extent to which patient perspectives and views were considered during the CPG development process, offering insight into the inclusivity of ADHD guideline formulation. Furthermore, we allocated at least one psychiatrist per CPG for appraisal, ensuring expert input during the appraisal process. Lastly, our study summarizes the clinical content available of each CPG to help clinicians and healthcare providers find and implement care options.

Our study also had several limitations. First, despite using a systematic process to identify all possible ADHD CPGs, we limited our review to English-language or English-translated CPGs, which may affect the generalizability of our findings. Future studies should include non-English CPGs to provide a more comprehensive understanding of current ADHD guidelines across geographical and cultural contexts.

Second, although the AGREE II instrument is a widely used and validated tool for appraising CPG quality, it has limitations. For example, the scoring system lacks specific guidelines on how to interpret scores between 2 and 6, which may result in subjective interpretations. This subjectivity can result in the inconsistency of item assessments across different appraisers. Additionally, the AGREE II instrument does not assess the strength of recommendations, meaning its scores do not represent the quality of evidence supporting those recommendations. For instance, the NICE CPG provides a full evidence review for each clinical question on their website, whereas the AAP and MAHTAS CPGs provide the development methodology without detailed accessible evidence reviews. Furthermore, no empirical data links specific AGREE II domain scores with specific implementation outcomes, leading to potential variations in interpreting CPG quality (24). Previous studies have reported that one-third of AGREE II users apply various thresholds for categorizing high- and low-quality CPGs (61, 62). To address these limitations, future studies could benefit from clearer guidance on scoring and more objective thresholds and criteria for interpreting domain scores, as well as incorporating tools like AGREE-REX to evaluate the strength of recommendations.

Finally, although we provide a summary of the recommendations for the diagnosis and management of ADHD and the quality of the included CPGs, clinicians and relevant stakeholders who wish to apply our findings should consider country-specific conditions, such as the availability of certain drugs, health insurance policies, and laws regarding the age requirements for psychostimulant use. These factors may influence the implementation of CPGs in routine clinical practice.

## Conclusion

Our systematic review revealed that only some of the included ADHD CPGs are either strongly recommended or recommended. Our findings highlight that domains 3 (rigor of development) and 5 (applicability) of the AGREE II instrument require particular attention to improve the quality of new ADHD CPGs. Future CPGs could be improved by involving methodological specialists in the CPG development process and using a systematic procedure and high-quality evidence. Furthermore, barriers and facilitators, additional CPG implementation tools, auditing, and monitoring will need to be addressed more effectively.

## Data Availability

The raw data supporting the conclusions of this article will be made available by the authors, without undue reservation.
