# Supplementary material for: Quality appraisal of clinical practice guidelines for attention deficit hyperactivity disorder: a systematic review using the appraisal of guidelines for research and evaluation (AGREE II) instrument
Source: Front Psychiatry. 2025 Jun 16;16:1576538. doi: 10.3389/fpsyt.2025.1576538 (PMC12206699; doi:10.3389/fpsyt.2025.1576538)
Supplement: Supplementary file 1 [file DataSheet1.pdf]

INPLASY PROTOCOL

To cite: Wahyu et al. A critical appraisal of clinical practice guidelines for the diagnosis & management of attention deficit hyperactivity disorder (ADHD): assessment using the Appraisal of Guidelines for Research and Evaluation (AGREE II) Instrument. Inplasy protocol 202280001. doi: 10.37766/inplasy2022.8.0001

Received: 01 August 2022

Published: 01 August 2022

Corresponding author:
Atsunori Sugimoto

sugimoto.pedpsy@gmail.com

Author Affiliation:
Department of Psychiatry, Niigata University, Niigata, Japan.

Support: **Niigata Prefectural Hospital.**

Review Stage at time of this submission: **Risk of bias assessment.**

A critical appraisal of clinical practice guidelines for the diagnosis & management of attention deficit hyperactivity disorder (ADHD): assessment using the Appraisal of Guidelines for Research and Evaluation (AGREE II) Instrument

Wahyu, MD[1]; Sugimoto, A[2]; Zain, E[3]; Parawansa, FBP[4]; Kasahara, H[5]; Yoshinaga, K[6]; Egawa, J[7]; Someya, T[8].

Review question / Objective: **• How many current practice guidelines on the diagnosis and management of attention deficit hyperactivity disorder (ADHD) are evaluable? • Which of the ADHD clinical practice guidelines recommendations released is the best based on the AGREE II instrument? • What advice do the guidelines have about the use of the following management: pharmacological & nonpharmacological treatment, side effects of drug therapy, termination of treatment, treatment of adverse effects, and monitoring & follow-up?**

Condition being studied: **This systematic review will focus on ADHD patients in children, adolescents, and/or adults. Although the results of a meta-analysis are not integrated, the qualitative evaluation of each CPG will be performed using an AGREE II instrument as an appraisal tool.**

INPLASY registration number: This protocol was registered with the International Platform of Registered Systematic Review and Meta-Analysis Protocols (INPLASY) on 01 August 2022 and was last updated on 01 August 2022 (registration number INPLASY202280001).

INTRODUCTION

Review question / Objective: **• How many current practice guidelines on the diagnosis and management of attention deficit hyperactivity disorder (ADHD) are evaluable?**

• Which of the ADHD clinical practice guidelines recommendations released is the best based on the AGREE II instrument?

• What advice do the guidelines have about the use of the following management: pharmacological & nonpharmacological

Wahyu et al. Inplasy protocol 202280001. doi:10.37766/inplasy2022.8.0001 Downloaded from https://inplasy.com/inplasy-2022-8-0001/

treatment, side effects of drug therapy, termination of treatment, treatment of adverse effects, and monitoring & follow-up?

Condition being studied: This systematic review will focus on ADHD patients in children, adolescents, and/or adults. Although the results of a meta-analysis are not integrated, the qualitative evaluation of each CPG will be performed using an AGREE II instrument as an appraisal tool.

METHODS

Search strategy: This systematic review will focus on published clinical practice guidelines for diagnosing and managing ADHD. Searches for studies or clinical practice guidelines will be conducted in accordance with PRISMA standards that are accessible and available on the PRISMA website (http://prisma-statement.org).

Participant or population: Clinical practice guidelines on ADHD children, adolescents, and/or adults.

Intervention: The subjects of the systematic review are CPGs, so no specific intervention will be performed. We will only consider CPGs that include diagnosis and management of ADHD. The criteria of the AGREE II instrument will be used to assess the methodological rigour and transparency in which a guideline is developed.

Comparator: Not applicable.

Study designs to be included: Clinical Practice Guidelines (CPGs).

Eligibility criteria: Inclusion Criteria: Topic: CPGs focused on the diagnosis and/or management of ADHD; Methods: Evidence-based Clinical Practice Guidelines (CPGs) involving recommendations or statements. The latest version of CPGs, and full-text accessibility; Status: Original sources; Languages: English or English translated CPGs; Authorship: CPGs issued or endorsed by national or international scientific societies or government organizations; Publisher/Issuer: Published by an organization/group authorship in a CPG database or peer-reviewed journal or organization that has the relevant authorities (such as the ministry of health, academic organization, etc.); Date of publication: Published between 2012-01-01 and 2021-12-31. Exclusion Criteria: CPGs with fewer than three authors; A relevant publication summarizing / reporting / reviewing the contents, or implementing the included original CPGs; Only focus on specific or specialized ADHD problems.

Information sources: 1. PubMed https://pubmed.ncbi.nlm.nih.gov/ 2. Google Scholar http://scholar.google.com/ 3. EBSCO DynaMed Plus (US) https://dynamed.ebscohost.com/ 4. American Agency for Healthcare Research and Quality's (AHRQ) National Guideline Clearinghouse (US) http://www.guidelines.gov 5. Guidelines International Network (GIN) http://www.g-i-n.net/library/international-guidelines-library 6. Scottish Intercollegiate Guidelines Network (SIGN; UK) http://www.sign.ac.uk/index.html 7. National Institute of Health and Care Excellence (NICE; UK) http://www.nice.org.uk/ 8. Australian National Health and Medical Research Council (NHMRC) 9. CPGs are published by national or international scientific societies or government organizations that have the relevant authorities (e.g. American Psychiatric Association, European Psychiatric Association, the Ministry of Health, etc). Additionally, we will also conduct internet searches through citation searching for relevant CPGs and CPGs published online only.

Main outcome(s): No meta-analysis will be performed in this study, but the only measurable outcome obtained in this study will be the quality of the clinical practice guidelines. This will be assessed by using the AGREE II instrument.
The AGREE II is comprised of 23 items organized into 6 quality domains:
1. Scope and purpose
2. Stakeholder involvement
3. Rigour of development

Wahyu et al. Inplasy protocol 202280001. doi:10.37766/inplasy2022.8.0001 Downloaded from https://inplasy.com/inplasy-2022-8-0001/

4. Clarity of presentation
5. Applicability; and
6. Editorial independence

Each of the 23 items targets various aspects of clinical practice guideline quality. The AGREE II also includes 2 final overall assessment items that require the appraiser to make overall judgments of the practice guidelines while considering how they rated the 23 items.

Additional outcome(s): None.

Data management: Inclusion and exclusion criteria will be applied to the title and abstract of each identified citation from database searching independently by three reviewers, settling disagreements through a discussion with a fourth reviewer. The same reviewers will find through citation/reference search and the internet to identify the other possible records which are not available in the database or previous searches. The full text will be obtained from papers that appear to meet the criteria and those for which a decision is not possible based on the information contained within the title and abstract alone. The full text of each paper will be assessed independently for inclusion by the same reviewers and disagreement will be resolved through discussion with the entire group by integrating each reviewer's opinion. The same team of reviewers will extract data from all eligible studies/clinical practice guidelines. Extracted data will include the CPGs title, developer organization, year of publication, country of publication, retrieved from, URL or DOI, and comments (if applicable) or other information which is relevant to 23 items on the AGREE II Instrument by utilizing the MY AGREE PLUS (online tool from AGREE II) that is accessible and available for free from AGREE II Enterprise website (http://www.agreetrust.org/). All assessors will independently do the scoring of all the CPG documents, as well as any supplementary files or links to web pages relating to the methodology or implementation tools of the guidelines. Furthermore, the assessors will give scores for every item on a 1-7 scale (1– strongly disagree to 7–strongly agree) and record the reasons for their scores in the discussion box for each question. In addition, the assessors will consider how the CPGs address the listed questions based on AGREE II instrument criteria using the MY AGREE PLUS platform. Finally, the standard AGREE domain score generates a final score in the form of a percentage (ranging from 0% to 100%) which will be automatically calculated by My AGREE PLUS following the equation provided by the AGREE II User Guide. If there will be a discrepancy in assessors' ratings, it will be handled by having a group discussion, and those who produce outlying scores will re-assess their ratings. Moreover, a PRISMA-style flowchart will be produced and reported to show the details of the study selection process, including reasons for exclusion.

Quality assessment / Risk of bias analysis: To reduce the risk of bias, when searching for potential CPGs and screening the included CPGs, the reviewers will use criteria based on the PIPOH framework. After that, at least three reviewers will also evaluate the CPGs included in this review independently by using the English version of the AGREE II instrument (via the online platform: MY AGREE PLUS) and will integrate the result to reduce the risk of bias. This instrument consists of 23 items grouped into six domains: scope and purpose, stakeholder involvement, the rigour of development, clarity and presentation, applicability, and editorial independence. Each reviewer will participate in an online course before the appraisal process to gain the same competency in evaluating CPGs using the AGREE II instrument.

Strategy of data synthesis: The results of our study will not be an integrated meta-analysis, but the qualitative evaluation of each CPG will be performed using an AGREE II instrument. In addition, for the CPGs that are eligible for inclusion, descriptive statistics will be generated and presented in a tabular format. Appraisal of Guidelines, Research and Evaluation II (AGREE II) instrument (https://www.agreetrust.org/resource-centre/agree-plus/) will be conducted online (MY

Wahyu et al. Inplasy protocol 202280001. doi:10.37766/inplasy2022.8.0001 Downloaded from https://inplasy.com/inplasy-2022-8-0001/

AGREE PLUS) for eligible CPGs. This widely used and validated tool assesses the methodological rigor and transparency in which a guideline is developed. There are 23 items in AGREE II, grouped into six categories: scope and purpose, stakeholder involvement, the rigour of development, clarity and presentation, applicability, and editorial independence. This instrument is described more in detail on the official website of AGREE Enterprise (www.agreetrust.org). An instructional manual is available on this website in which detailed directions are given on how to score and where to find the relevant guidelines to score each item for each domain. In addition, CPG quality scores will be calculated for each of the six AGREE II domains using an online platform. No additional software, tools, or statistical tests (apart from the online platform) will be needed to perform AGREE II.

Subgroup analysis: None.

Sensitivity analysis: None.

Language restriction: English.

Country(ies) involved: Japan.

Other relevant information: Detailed information related to the funding source: Niigata Prefectural Hospital Bureau Commissioned Research Fund(156195-J15F0001).

Keywords: attention-deficit/hyperactivity disorder, ADHD, guideline, practice guideline, clinical practice guideline, practice parameter, guidance, recommendations, systematic review, AGREE II.

Dissemination plans: We will submit the results of this study to peer-reviewed scientific journals. When a treatise is accepted and published in a scientific journal, it will be published on our organization's website (http://www.niigata-dp.org/).

Contributions of each author:
Author 1 - Muhammad Dwi Wahyu.
Author 2 - Atsunori Sugimoto.
Author 3 - Ekachaeryanti Zain.
Author 4 - Faisal Budisasmita Paturungi Parawansa.
Author 5 - Hiroyuki Kasahara.
Author 6 - Kiyohiro Yoshinaga.
Author 7 - Jun Egawa.
Author 8 - Toshiyuki Someya.

Conflicts of interest: Sugimoto, A has received research funding or honoraria from the following organizations in the last 5 years; Developmental Science Research and Education Center, Taiju Life and Welfare Foundation, Kawano Pediatric Medical Scholarship Foundation, Shimadzu Corporation, Takeda Pharmaceutical Co., Ltd., Nobel Pharma Co., Ltd., Shionogi Co., Ltd., Mochida Pharmaceutical Co., Ltd. The other authors declare no competing interest.
Egawa, J has received research funding or honoraria from the following organizations in the last 5 years; Takeda Pharmaceutical Co. Ltd., Shionogi Co. Ltd.
Someya, T has received research funding or honoraria from the following organizations in the last 5 years; Astellas Pharma Inc., Eli Lilly Japan K.K., Janssen Pharmaceutical K.K., Mitsubishi Tanabe Pharma Co., Mochida Pharmaceutical Co., Ltd., MSD K.K., Novartis Pharma K.K., Otsuka Pharmaceutical Co., Ltd., Shionogi Co., Ltd., Sumitomo Pharma Co., Ltd.

Wahyu et al. Inplasy protocol 202280001. doi:10.37766/inplasy2022.8.0001 Downloaded from https://inplasy.com/inplasy-2022-8-0001/
