# Supplementary material for: Quality appraisal of clinical practice guidelines for attention deficit hyperactivity disorder: a systematic review using the appraisal of guidelines for research and evaluation (AGREE II) instrument
Source: Front Psychiatry. 2025 Jun 16;16:1576538. doi: 10.3389/fpsyt.2025.1576538 (PMC12206699; doi:10.3389/fpsyt.2025.1576538)
Supplement: Supplementary file 2 [file DataSheet2.pdf]

Table S1. PRISMA checklist

Section and Topic	Item #	Checklist item	Location where item is reported
**TITLE**			
Title	1	Identify the report as a systematic review.	Page 1 Lines 1–3 TITLE
**ABSTRACT**			
Abstract	2	See the PRISMA 2020 for Abstracts checklist.	Page 1 Lines 20–45 ABSTRACT
**INTRODUCTION**			
Rationale	3	Describe the rationale for the review in the context of existing knowledge.	Pages 3–4 Lines 50–82 INTRODUCTION
Objectives	4	Provide an explicit statement of the objective(s) or question(s) the review addresses.	Page 4 Lines 83–86 INTRODUCTION (This systematic review aims to…)
**METHODS**			
Eligibility criteria	5	Specify the inclusion and exclusion criteria for the review and how studies were grouped for the syntheses.	Pages 4–5 Lines 95–133 METHODS (Eligibility Criteria, PIPOH Framework)
Information sources	6	Specify all databases, registers, websites, organisations, reference lists and other sources searched or consulted to identify studies. Specify the date when each source was last searched or consulted.	Pages 6–7 Lines 135–150 METHODS (Search Strategy); Supplementary Files Tables S2-S4. Search Strategy
Search strategy	7	Present the full search strategies for all databases, registers and websites, including any filters and limits used.	Pages 6–7 Lines 135-150 METHODS (Search Strategy)
Selection process	8	Specify the methods used to decide whether a study met the inclusion criteria of the review, including how many reviewers screened each record and each report retrieved, whether they worked independently, and if applicable, details of automation tools used in the process.	Page 7 Lines 152–159 METHODS (Study Selection Process)
Data collection process	9	Specify the methods used to collect data from reports, including how many reviewers collected data from each report, whether they worked independently, any processes for obtaining or confirming data from study investigators, and if applicable, details of automation tools used in the process.	Page 7 Lines 161–165 METHODS (Data Extraction)
Data items	10a	List and define all outcomes for which data were sought. Specify whether all results that were compatible with each outcome domain in each study were sought (e.g. for all measures, time points, analyses), and if not, the methods used to decide which results to collect.	Pages 7–8 Lines 161–189 METHODS (Data Extraction, Guidelines Quality Assessment, Rating of AGREE II domains)<br>Because the systematic review targets guidelines, the results are recommendations rather than numerical variables. Although meta-analysis was not possible for this reason, we summarized the recommendations of each guideline and evaluated the quality of each guideline using AGREE-II.
	10b	List and define all other variables for which data were sought (e.g. participant and intervention characteristics, funding sources). Describe any assumptions made about any missing or unclear information.	Pages 5–6 Lines 119–133 PIPOH Framework; Page 7 Lines 161–165 METHODS (Data Extraction)<br>Because the systematic review targets guidelines, the results are recommendations rather than numerical variables. Although meta-analysis was not possible for this reason, we summarized the recommendations of each guideline and evaluated the quality of each guideline using AGREE-II.
Study risk of bias	11	Specify the methods used to assess risk of bias in the included studies, including	Supplementary Files 1. Study Protocol; `

Section and Topic	Item #	Checklist item	Location where item is reported
assessment		details of the tool(s) used, how many reviewers assessed each study and whether they worked independently, and if applicable, details of automation tools used in the process.	
Effect measures	12	Specify for each outcome the effect measure(s) (e.g. risk ratio, mean difference) used in the synthesis or presentation of results.	Pages 8–9 Lines 191–217 METHODS (Data Synthesis and Analyses)
Synthesis methods	13a	Describe the processes used to decide which studies were eligible for each synthesis (e.g. tabulating the study intervention characteristics and comparing against the planned groups for each synthesis (item #5)).	Pages 8–9 Lines 191–217 METHODS (Data Synthesis and Analyses)
	13b	Describe any methods required to prepare the data for presentation or synthesis, such as handling of missing summary statistics, or data conversions.	Page 8 Lines 183–189 METHODS (Rating of AGREE II domains)
	13c	Describe any methods used to tabulate or visually display results of individual studies and syntheses.	Pages 8–9 Lines 191–217 METHODS (Data Synthesis and Analyses: IBM SPSS Statistics Version 28)
	13d	Describe any methods used to synthesize results and provide a rationale for the choice(s). If meta-analysis was performed, describe the model(s), method(s) to identify the presence and extent of statistical heterogeneity, and software package(s) used.	Pages 8–9 Lines 191–217 METHODS (Data Synthesis and Analyses: CPG Classification strongly recommended, recommended, and not recommended)
	13e	Describe any methods used to explore possible causes of heterogeneity among study results (e.g. subgroup analysis, meta-regression).	Pages 8–9 Lines 191–217 METHODS (Data Synthesis and Analyses: Interrater reliability with ICC analysis)
	13f	Describe any sensitivity analyses conducted to assess robustness of the synthesized results.	Pages 8–9 Lines 191–217 METHODS (Data Synthesis and Analyses: Interrater reliability with ICC analysis)
Reporting bias assessment	14	Describe any methods used to assess risk of bias due to missing results in a synthesis (arising from reporting biases).	Page 7 Lines 152–159 METHODS (Study Selection Process); Pages 8–9 Lines 191–217 METHODS (Data Synthesis and Analyses)
Certainty assessment	15	Describe any methods used to assess certainty (or confidence) in the body of evidence for an outcome.	Pages 7–9 Lines 167–217 METHODS (Guidelines Quality Assessment, Rating of AGREE II domains, Data Synthesis and Analyses)
**RESULTS**			
Study selection	16a	Describe the results of the search and selection process, from the number of records identified in the search to the number of studies included in the review, ideally using a flow diagram.	Page 10 Lines 219–230 RESULTS (Included ADHD CPGs); Figure 1. PRISMA Flow Diagram
	16b	Cite studies that might appear to meet the inclusion criteria, but which were excluded, and explain why they were excluded.	Page 10 Lines 219–230 RESULTS (Included ADHD CPGs); Supplementary Files Table S5. List of Excluded Studies
Study characteristics	17	Cite each included study and present its characteristics.	Pages 10–15 Line 219–365 RESULTS (Included ADHD CPGs, CPGs characteristics, Review of CPG Recommendation); Table 1. General characteristics of ADHD CPGs; Table 3. Recommendations of the reviewed CPGs for the non-pharmacological management of ADHD; Table 4. Recommendations of the reviewed CPGs on the pharmacological management of ADHD
Risk of bias in studies	18	Present assessments of risk of bias for each included study.	Pages 16–17 Lines 385–397 RESULTS (Interrater Reliability); Figure 3. Interrater Agreement of AGREE II Domain Scores; Supplementary Table S7. Interrater reliability using the ICC for AGREE II domain scores

Section and Topic	Item #	Checklist item	Location where item is reported
Results of individual studies	19	For all outcomes, present, for each study: (a) summary statistics for each group (where appropriate) and (b) an effect estimate and its precision (e.g. confidence/credible interval), ideally using structured tables or plots.	Pages 16–17 Lines 367–397 RESULTS (Quality Appraisal based on AGREE II domains, Quality of Each CPG, Interrater Reliability); Figure 2. AGREE II Domain Scores for Included ADHD CPGs; Figure 3. Interrater Agreement of AGREE II Domain Scores; Supplementary Table S6. Quality of ADHD CPGs based on domain score using the AGREE II instrument; Supplementary Table S7. Interrater reliability using the ICC for AGREE II domain scores
Results of syntheses	20a	For each synthesis, briefly summarise the characteristics and risk of bias among contributing studies.	Pages 16–17 Lines 378–397 RESULTS (Quality of Each CPG and Interrater Reliability); Figure 2. AGREE II Domain Scores for Included ADHD CPGs; Figure 3. Interrater Agreement of AGREE II Domain Scores; Supplementary Table S6. Quality of ADHD CPGs based on domain score using the AGREE II instrument; Supplementary Table S7. Interrater reliability using the ICC for AGREE II domain scores
	20b	Present results of all statistical syntheses conducted. If meta-analysis was done, present for each the summary estimate and its precision (e.g. confidence/credible interval) and measures of statistical heterogeneity. If comparing groups, describe the direction of the effect.	Pages 16–17 Lines 367–397 RESULTS (Quality Appraisal based on AGREE II domains, Quality of Each CPG, Interrater Reliability); Figure 2. AGREE II Domain Scores for Included ADHD CPGs; Figure 3. Interrater Agreement of AGREE II Domain Scores; Supplementary Table S6. Quality of ADHD CPGs based on domain score using the AGREE II instrument; Supplementary Table S7. Interrater reliability using the ICC for AGREE II domain scores
	20c	Present results of all investigations of possible causes of heterogeneity among study results.	Pages 16–17 Lines 385–397 RESULTS (Interrater Reliability); Figure 3. Interrater Agreement of AGREE II Domain Scores; Supplementary Table S7. Interrater reliability using the ICC for AGREE II domain scores
	20d	Present results of all sensitivity analyses conducted to assess the robustness of the synthesized results.	Page 8 Line 289-302 RESULTS (Interrater Reliability); Figure 3. Interrater Agreement of AGREE II Domain Scores; Supplementary Table S7. Interrater reliability using the ICC for AGREE II domain scores
Reporting biases	21	Present assessments of risk of bias due to missing results (arising from reporting biases) for each synthesis assessed.	N/A<br>Because the systematic review targets guidelines, the results are recommendations rather than numerical variables. Although meta-analysis was not possible for this reason, we summarized the recommendations of each guideline and evaluated the quality of each guideline using AGREE-II.
Certainty of evidence	22	Present assessments of certainty (or confidence) in the body of evidence for each outcome assessed.	Pages 16–17 Lines 367–397 RESULTS (Quality Appraisal based on AGREE II domains, Quality of Each CPG, Interrater Reliability); Figure 2. AGREE II Domain Scores for Included ADHD CPGs; Figure 3. Interrater Agreement of AGREE II Domain Scores; Supplementary Table S6. Quality of ADHD CPGs based on domain score using the AGREE II instrument; Supplementary Table S7. Interrater reliability using the ICC for AGREE II domain scores
**DISCUSSION**			
Discussion	23a	Provide a general interpretation of the results in the context of other evidence.	Pages 17–20 Lines 399–485 DISCUSSION (Comparison of CPG

Section and Topic	Item #	Checklist item	Location where item is reported
			recommendation; Comparison by Each AGREE II Domain)
	23b	Discuss any limitations of the evidence included in the review.	Pages 18–20 Lines 438–485 DISCUSSION (Comparison by Each AGREE II Domain)
	23c	Discuss any limitations of the review processes used.	Pages 20–22 Lines 487–522 DISCUSSION (Strengths and Limitations of The Study)
	23d	Discuss implications of the results for practice, policy, and future research.	Pages 20–22 Lines 487–522 DISCUSSION (Strengths and Limitations of The Study)
**OTHER INFORMATION**			
Registration and protocol	24a	Provide registration information for the review, including register name and registration number, or state that the review was not registered.	Page 2 Line 45 INPLASY registration number INPLASY202280001; Supplementary File 1. Study Protocol
	24b	Indicate where the review protocol can be accessed, or state that a protocol was not prepared.	Page 2 Line 45 INPLASY registration number INPLASY202280001; Supplementary File 1. Study Protocol
	24c	Describe and explain any amendments to information provided at registration or in the protocol.	N/A<br>No amendment has been made
Support	25	Describe sources of financial or non-financial support for the review, and the role of the funders or sponsors in the review.	Page 24 Lines 570–574 Funding
Competing interests	26	Declare any competing interests of review authors.	Page 23 Line 550–558 Conflict of Interest
Availability of data, code and other materials	27	Report which of the following are publicly available and where they can be found: template data collection forms; data extracted from included studies; data used for all analyses; analytic code; any other materials used in the review.	Supplementary File 1. Study protocol; Supplementary Files Table S1. Preferred Reporting Items for Systematic Reviews and Meta-Analyses (PRISMA 2020) checklist; Supplementary Table S2. Search strategy for PubMed; Supplementary Table S3. Search strategy for Google Scholars; Supplementary Table S4. Websites of guideline databases; Supplementary Table S5. List of excluded studies; Supplementary Table S6. Quality of ADHD CPGs based on domain score using the AGREE II instrument; Supplementary Table S7. Interrater reliability using the ICC for AGREE II domain scores

From: Page MJ, McKenzie JE, Bossuyt PM, Boutron I, Hoffmann TC, Mulrow CD, et al. The PRISMA 2020 statement: an updated guideline for reporting systematic reviews. BMJ 2021;372:n71. doi: 10.1136/bmj.n71

For more information, visit: http://www.prisma-statement.org/
