# Supplementary material for: Quality appraisal of clinical practice guidelines for attention deficit hyperactivity disorder: a systematic review using the appraisal of guidelines for research and evaluation (AGREE II) instrument
Source: Front Psychiatry. 2025 Jun 16;16:1576538. doi: 10.3389/fpsyt.2025.1576538 (PMC12206699; doi:10.3389/fpsyt.2025.1576538)
Supplement: Supplementary file 3 [file DataSheet3.pdf]

Table S2. Search strategy for PubMed

	Search term
#1	"attention-deficit/hyperactivity disorder" [Mesh] OR ADHD [Mesh]
#2	"attention-deficit/hyperactivity disorder" [Title/Abstract] OR ADHD [Title/Abstract]
#3	guideline* [Title/Abstract] OR "practice guideline" [Title/Abstract] OR "clinical practice guideline" [Title/Abstract] OR "practice parameter" [Title/Abstract] OR guidance [Title/Abstract] OR recommendations [Title/Abstract]
#4	#2 AND #3
#5	Filters applied: Publication date from 1/1/2012 to 31/12/2021, Humans, Guideline

Table S3. Search strategy for Google Scholars

	Search term
#1	"attention-deficit/hyperactivity disorder" AND "guideline" after:2012 before:2022
#2	"attention-deficit/hyperactivity disorder" AND "clinical practice guideline" after:2012 before:2022
#3	"ADHD" AND "practice guideline" after:2012 before:2022
#4	""ADHD" AND "guidance" after:2012 before:2022

Table S4. Websites of guideline databases

Guideline databases	Website
EBSCO DynaMed Plus	https://dynamed.ebscohost.com
AHRQ	http://www.guidelines.gov
GIN	http://www.g-i-n.net/library/international-guidelines-library
SIGN	http://www.sign.ac.uk/index.html
NICE	http://www.nice.org.uk
NHMRC	https://www.nhmrc.gov.au
APA	https://www.apa.org
EPA	https://www.europsy.net
Saudi ADHD society	https://cpg.adhd.org.sa/en/
UMHS	https://www.uofmhealth.org
Kementerian Kesihatan Malaysia	https://www.moh.gov.my
Ministry of Health of Singapore	https://www.moh.gov.sg

EBSCO, Evidence-Based Medicine Clinical Outcomes; AHRQ, Agency for Healthcare Research and Quality; GIN, Guidelines International Network; SIGN, Scottish Intercollegiate Guidelines Network; NICE, National Institute for Health and Care Excellence; NHMRC, National Health and Medical Research Council; APA, American Psychiatric Association; EPA, European Psychiatric Association; ADHD, Attention Deficit Hyperactivity Disorders; UMHS, University of Michigan Health System
