# Supplementary material for: Quality appraisal of clinical practice guidelines for attention deficit hyperactivity disorder: a systematic review using the appraisal of guidelines for research and evaluation (AGREE II) instrument
Source: Front Psychiatry. 2025 Jun 16;16:1576538. doi: 10.3389/fpsyt.2025.1576538 (PMC12206699; doi:10.3389/fpsyt.2025.1576538)
Supplement: Supplementary file 4 [file DataSheet4.pdf]

Table S5. List of excluded studies

(After duplicates records, published before 2012, not identified as CPG are excluded)

No	Study Title	Year	Country	Organization	Reasons for Exclusion
1	Society for Developmental and Behavioral Pediatrics Clinical Practice Guideline for the Assessment and Treatment of Children and Adolescents with Complex Attention-Deficit/Hyperactivity Disorder	2020	USA	Society for Developmental and Behavioral Pediatrics (SDBP)	Fit into exclusion criteria "specific or specialized ADHD"
2	The Canadian Network for Mood and Anxiety Treatments (CANMAT) task force recommendations for the management of patients with mood disorders and comorbid attention-deficit/hyperactivity disorder.	2012	Canada	CANMAT	Not identified as CPG *(Review article)* and fit into exclusion criteria "specific or specialized ADHD"
3	Practice advisory: The utility of EEG theta/beta power ratio in ADHD diagnosis, Report of the Guideline Development, Dissemination, and Implementation Subcommittee of the American Academy of Neurology	2016	USA	American Academy of Neurology	Not identified as CPG *(Review article)* and fit into exclusion criteria "specific or specialized ADHD"
4	Clinical Practice Guidelines for the Assessment and Management of Attention-Deficit/Hyperactivity Disorder	2019	India	Department of Psychiatry, Postgraduate Institute of Medical Education and Research, Chandigarh, India	This guideline was not developed by the Indian Psychiatric Society (a national or international scientific organization)
5	Practitioner Review: Current best practice in the management of adverse events during treatment with ADHD medications in children and adolescents	2013	USA	Association for Child and Adolescent Mental Health (ACAMH)	Not identified as CPG focused on the diagnosis and/or management of ADHD *(Practitioner review)*
6	Expert Recommendation: contributions to the clinical practice of the new prodrug lisdexamfetamine dimesylate (LDX) in the treatment of attention deficit hyperactivity disorder (ADHD)	2014	Spain	Spanish Society of Primary Care Pediatrics (AEPAP), Spanish Society of Pediatric Neurology (SENEP), Spanish Society of Out-hospital Pediatrics and Primary Care (SEPEAP)	Not identified as CPG which is focusing on diagnosis and management of ADHD (only expert recommendation of lisdexamfetamine dimesylate (LDX)

7	The Guideline of Diagnosis and Treatment of Attention-Deficit Hyperactivity Disorder: Developed by ADHD Translational Research Center	2016	South Korea	The Korean Academy of Child and Adolescent Psychiatry	Full text in Korean language, only tables and figures available in English
8	Treatment Receipt and Outcomes from a Clinic Employing the Attention-Deficit/Hyperactivity Disorder Treatment Guideline of the Children's Medication Algorithm Project	2014	Canada	(Non-Institutional)	Not match these 2 eligibility criteria:<br>• Not identified as CPG<br>• Not published by a national or international scientific organization
9	Management of attention deficit hyperactivity disorder in children and adolescents: A national clinical guideline	2020	Iran	Iran University of Medical Sciences	• Full text in Arabian language<br>• This guideline was not issued or endorsed by national or international scientific societies or government organizations
10	Attention-Deficit/Hyperactivity Disorder: AAP Updates Guideline for Diagnosis and Management	2020	USA	American Family Physician	This is a review article summarizing/reporting/reviewing the contents of AAP guidelines.
11	Clinical practice guideline on pharmacological and psychological management of adult patients with attention deficit and hyperactivity disorder and comorbid substance use.	2022	Spain	Servicio Gallego de Salud (SERGAS) and Sociedad Es- pañola de Psiquiatría Biológica (SEPB).	This guideline focus on the management of ADHD with comorbid substance use disorder (SUD).
12	Management approaches for ADHD during the COVID-19 virus pandemic: guidance from the European ADHD Guidelines Group (EAGG)	2020	USA, UK, and other European Countries	European ADHD Guidelines Group (EAGG)	This is a review article that describes ADHD management during the COVID-19 pandemic which is out of our scope.
13	ADHD in Children and Adolescents: A Good Practice Guidance	2012	UK	National Paediatric ADHD Interest Group	This study was written only by 1 author (single authorship) and not identified as CPG (*review article*)
14	Improving Interactions between Teachers and Parents of Children with ADHD, Family Research and Teacher Recommendations	2015	UK	The International Dyslexia Association	This is a review article that describes the family and teachers' role for children with ADHD (Not identified as CPG)
15	Recommendations for the Treatment of Attention-Deficit/Hyperactivity Disorder (ADHD)	2017	USA	-	This record reviews the contents of AAP guidelines and was written only by 2 authorships.

16	Drug Regimen Individualization for Attention-Deficit/Hyperactivity Disorder: Guidance for Methylphenidate and Dexmethylphenidate Formulations	2019	USA	-	This is a review article that describes drug regimen formulation.
17	Guidance for identification and treatment of individuals with attention deficit/ hyperactivity disorder and autism spectrum disorder based upon expert consensus	2020	UK & USA	The United Kingdom ADHD Partnership (UKAP)	This guideline focus on the management of ADHD with co-occurring autism spectrum disorder (ASD).
18	Females with ADHD: An expert consensus statement taking a lifespan approach providing guidance for the identification and treatment of attention-deficit/ hyperactivity disorder in girls and women	2020	UK	The United Kingdom ADHD Partnership (UKAP)	This guideline focus on specific ADHD condition *(Females with ADHD)*
19	Guideline for Screening, Diagnosis and Treatment of ADHD in Adults with Substance Use Disorders	2014	Belgium & The Netherlands	Belgian Center for Evidence Based Medicine (CEBAM)	This guideline focus on the management of adult ADHD with comorbid substance use disorder (SUD)."
20	Behavior Therapy as First-Line Treatment for Preschool-Aged Children with ADHD	2021	USA	Agency for Healthcare Research and Quality [AHRQ]	Not identified as CPG (*draft measure)*
21	Accurate ADHD Diagnosis	2014	USA	Agency for Healthcare Research and Quality [AHRQ]	Not identified as CPG (*draft measure)*
22	Attention Deficit Hyperactivity Disorder Performance Measurement Set	2012	USA	ADHD Expert Work Group and Agency for Healthcare Research and Quality [AHRQ]	Not identified as CPG (*draft measure)*
23	Measure: Attention Deficit Hyperactivity Disorder (ADHD) – Measure of Chronic Care Followup	2015	USA	AHRQ-CMS Pediatric Quality Measures Program (PQMP)	Not identified as CPG (*draft measure)*
24	Measure: Accurate ADHD Diagnosis	2014	US	AHRQ-CMS Pediatric Quality Measures Program (PQMP)	Not identified as CPG (*draft measure)*
25	A Guide to Getting an ADHD Assessment as an adult in Scotland	2019	Scotland (UK)	Scottish ADHD Coalition	Not identified as CPG *(public information)*
26	Medications for Attention Deficit Hyperactivity Disorder (ADHD) in Children	2022	USA & Netherlands	-	Cannot access the full text

27	Evidence Based Clinical Practice Guideline for Management of Attention Deficit Hyperactivity Disorder ADHD in Saudi Arabia.	2020	Saudi Arabia	Saudi ADHD Society	This guideline adopted the contents of the NICE guideline.
28	Some Guidelines for Conducting Research in Applied Behavioral Pharmacology	2013	USA	-	Not identified as CPG focuses on the diagnosis and/or management of ADHD, but guidelines for conducting research in applied behavioral pharmacology.
29	Korean Practice Parameter for Adult Attention-Deficit/Hyperactivity Disorder	2020	South Korea	The Adult ADHD Special Interest Study Group (the Korean of Child and Adolescent Psychiatry)	This guideline is not issued or endorsed by national or international scientific societies or government organizations)
30	ADHD in school-aged youth: Management and special treatment considerations in the primary care setting	2016	USA	-	Not match these 3 eligibility criteria :<br>• Not identified as CPG *(Review article)*<br>• Single authorship<br>• Not issued or endorsed by national or international scientific societies or government organizations
31	Shared care guideline for the use of Methylphenidate, Dexamfetamine, Lisdexamfetamine dimesylate & Atomoxetine for the management of Attention Deficit Hyperactivity Disorder (ADHD) in adults patients	2018	UK	NHS Foundation Trust	This record reviews the contents of the NICE guideline.
32	ADHD management during the COVID-19 pandemic: guidance from the European ADHD Guidelines Group	2020	USA, UK, and other European Countries	European ADHD Guidelines Group (EAGG)	This is a review article that describes ADHD management during the COVID-19 pandemic which is out of our scope.
33	Recommendations for the transition of patients with ADHD from child to adult healthcare services: a consensus statement from the UK adult ADHD network	2016	UK	UK Adult ADHD Network (UKAAN)	This is a guideline that was not focusing on the assessment/management of ADHD, instead of only transitioning of patients with ADHD condition.
34	Starting ADHD medications during the COVID-19 pandemic: recommendations from the European ADHD Guidelines Group	2020	UK & Australia	European ADHD Guidelines Group (EAGG)	This record is an addendum of the European ADHD Guidelines Group (EAGG) on starting attention-deficit hyperactivity

					disorder (ADHD) during COVID-19.
35	Recommendations for occupational therapy interventions for adults with ADHD: a consensus statement from the UK adult ADHD network	2021	UK, Singapore, & USA	UK Adult ADHD Network	This is a guideline that was not focusing on the assessment/management of ADHD, instead of occupational therapy interventions for adults with ADHD.
36	Assessment and Management of Sleep<br>Problems in Youths With Attention-Deficit/Hyperactivity Disorder	2013	USA	AMERICAN ACADEMY OF CHILD & ADOLESCENT PSYCHIATRY	This is a guideline that was not focused on the assessment/management of ADHD, but instead on sleep problems in youth with ADHD.
37	Outside the Box: Rethinking ADD/ADHD in Children and Adults A Practical Guide	2017	USA	-	Not match these 2 eligibility criteria:<br>• Single authorship<br>• This is a book and not published by an organization/group authorship
38	A long version of the interdisciplinary evidence- and consensus-based (S3) guideline “Attention-Deficit/Hyperactivity Disorder (ADHD) in children, adolescents and adults”	2020	Germany	DGKJP<br>DGPPN<br>Deutsche Gesellschaft	This guideline only provides the English version of the recommendation (long version), however, the other file such as the summary document and methodology development only available in Germany.
39	Prescribing Guidelines for Attention-Deficit/Hyperactivity Disorder (ADHD)	2022	USA	Nationwide Children’s Hospital	The information related to the number of the author is unavailable.
40	Attention-Deficit/Hyperactivity Disorder in Children and Teens: What You Need to Know	2021	USA	National Institutes of Health (NIH)	This record is not identified as CPG.
41	Management of Pediatric Attention Deficit & Hyperactivity Disorder (ADHD) Clinical Practice Guideline	2021	USA	MedStar Health	• The information related to the number of the author is unavailable.<br>• This record is not issued or endorsed by national or international scientific societies or government organizations.
42	ADHD Parents Medication Guide	2013	USA	American Academy of Child<br>& Adolescent Psychiatry and American Psychiatric Association	This record is not identified as CPG.
43	Clinical Practice Guideline for Patients with Attention-Deficit/Hyperactivity Disorder	2020	USA	Magellan Health	This guideline is not published in the CPG database or peer-reviewed journal or organization that has the relevant authorities

					(such as the ministry of health, academic organization, etc.)
44	Attention Deficit Hyperactivity Disorder (ADHD) In Children and Adults	2020	USA	WADA – World Anti-Doping Agency	Not match these 2 eligibility criteria:<br>• Not identified as CPG<br>• The information about the number of authors is unavailable.<br>• This record is not published by a national or international scientific organization
45	Guidelines for the Pharmacological Management of Attention Deficit Hyperactivity Disorder (ADHD) in Children, Young People and Adults	2019	UK	Hertfordshire Partnership University NHS Foundation Trust (HPFT)	Not match these 2 eligibility criteria:<br>• This guideline reviewed the contents of the NICE and BAP guidelines.<br>• The information related to the number of the author is unavailable.
46	Guideline for Evaluation and Treatment of Attention-Deficit/Hyperactivity Disorder (ADHD) in adults	2017	USA	San Francisco Health Network Behavioral Health Services Medication Use Improvement Committee	Not match these 2 eligibility criteria:<br>• Single authorship<br>• This guideline reviewed the contents of the NICE and BAP guidelines.
47	Quality Improvement Guidelines Attention-Deficit Hyperactivity Disorder (ADHD)	2020	USA	Baylor Scott & White	Not match these 2 eligibility criteria:<br>• This record is not issued or endorsed by national or international scientific societies or government organizations.<br>• The information related to the number of the author is unavailable.
48	Good Clinical Practice in the recognition and treatment of ADHD in adults with substance use dependence	2016	Belgium	Vlaams expertisecentrum Alcohol en andere Drugs (VUD) & Forum voor Verslavingsgeneeskunde Vrije Universiteit Brussel (VUB)	Not match these 2 eligibility criteria:<br>• This guideline focus on the management of adult ADHD with comorbid substance use disorder (SUD).<br>• Only 2 authors
49	Clinical Guidelines for the Pharmacologic Treatment of Attention Deficit and Hyperactivity Disorder (ADHD)	2018	USA	CBH - Community Behavioral Health	Not match these 2 eligibility criteria:<br>• Not identified as CPG

					• Not published by a national or international scientific organization
50	Attention Deficit Hyperactivity Disorder (ADHD) Guideline for the treatment and care of children and young people with ADHD	2015	UK	Wirral University Teaching Hospital NHS Foundation Trust (WUTH)	Not match these 2 eligibility criteria:<br>• This guideline reviewed the contents of the NICE and AAP guideline.<br>• Single authorship (Only 1 author)
51	ADHD in adults: good practice guidelines ; Royal College of Psychiatrists in Scotland	2017	UK	Royal College of Psychiatrists Scotland	This guideline summarized NICE, BAP, and the European consensus statement.
52	The Australian evidence-based clinical practice guideline for attention deficit hyperactivity disorder	2023	Australia	The Australian ADHD Professionals Association (AADPA)	This guideline adopted the contents of the NICE guideline.

ADHD, Attention Deficit Hyperactivity Disorder*;* *AAP*, American Academy of Pediatrics; *COVID-19*, Coronavirus Disease 2019*;* *DGKJP,* Die Klinik für Kinder- und Jugendpsychiatrie/Pschotherapie; *DGPPN,* Deutsche Gesellschaft für Psychiatrie und Psychotherapie, Psychosomatik und Nervenheilkunde e. V; *NICE*, National Institute of Health and Care Excellence; *BAP*, the British Association for Psychopharmacology; *UK*, United Kingdom; *USA*, United States of America
