# Supplementary material for: Quality appraisal of clinical practice guidelines for attention deficit hyperactivity disorder: a systematic review using the appraisal of guidelines for research and evaluation (AGREE II) instrument
Source: Front Psychiatry. 2025 Jun 16;16:1576538. doi: 10.3389/fpsyt.2025.1576538 (PMC12206699; doi:10.3389/fpsyt.2025.1576538)
Supplement: Supplementary file 5 [file DataSheet5.pdf]

Table S6. Quality of ADHD CPGs based on domain score using the AGREE II instrument

No	Guideline	Domains using the AGREE II instrument											
		Scope and Purpose	Stakeholder Involvement	Rigour of Development	Clarity of Presentation	Applicability	Editorial Independence	OA 1	OA 2	Domain score			Recommendations
										≤ 30	31–59	≥ 60	
1	AAP	83%	56%	78%	89%	40%	75%	72%	Yes - 1, Yes with modifications - 2, No - 0	0	2	4	SR (+)
2	SMOH	46%	41%	26%	57%	39%	0%	39%	Yes - 0, Yes with modifications - 1, No - 2	2	4	0	NR (-)
3	CADDRA	69%	70%	47%	67%	53%	78%	61%	Yes - 1, Yes with modifications - 1, No - 1	0	2	4	R (+)
4	NICE	98%	89%	92%	93%	74%	78%	89%	Yes - 3, Yes with modifications - 0, No - 0	0	0	6	SR (++)
5	NHMRC	65%	65%	47%	76%	31%	78%	56%	Yes - 1, Yes with modifications - 2, No - 0	0	2	4	R (+)
6	MAHTAS	87%	81%	74%	83%	53%	72%	78%	Yes - 1, Yes with modifications - 2, No - 0	0	1	5	SR (++)
7	UMHS	63%	52%	60%	87%	69%	61%	67%	Yes - 2, Yes with modifications - 1, No - 0	0	1	5	R (+)
8	ICSI	80%	67%	60%	70%	53%	86%	67%	Yes - 1, Yes with modifications - 2, No - 0	0	1	5	R (+)
9	IAP	54%	24%	22%	63%	28%	33%	44%	Yes - 1, Yes with modifications - 1, No - 1	3	2	1	NR (-)
10	BAP	61%	48%	37%	63%	29%	53%	44%	Yes - 1, Yes with modifications - 0, No - 2	1	3	2	R (+)
11	EPA	56%	39%	19%	63%	28%	50%	44%	Yes - 1, Yes with modifications - 0, No - 2	2	3	1	NR (-)
	Mean ± SD	69.27 ± 15.9	57.45 ± 19.3	51.09 ± 24.1	73.73 ± 12.5	45.18 ± 16.4	60.36 ± 25.5	60.09 ± 16.2					
	Median	65	56	47	70	40	72	61					

	Shapiro-Wilk *p*-value	0.827	0.994	0.692	0.266	0.124	0.034	0.483	

ADHD, attention deficit hyperactivity disorder; AGREE II, Appraisal of Guidelines for Research and Evaluation Instrument Version II; OA, overall assessment; AAP, American Academy of Pediatrics; SMOH, Singapore Ministry of Health; CADDRA, Canadian ADHD Resource Alliance; NICE, National Institute of Health and Care Excellence; NHMRC, National Health Medical Research Center; MAHTAS, Malaysian Health Technology Assessment Section; UMHS, University of Michigan Health System; ICSI, Institute of Clinical System Improvement; IAP, Indian Academy of Pediatrics; BAP, British Association for Psychopharmacology; EPA, European Psychiatric Association; SD, standard deviation; SR, strong recommendation; R, recommendation; NR, not recommended
