# Supplementary material for: Quality appraisal of clinical practice guidelines for attention deficit hyperactivity disorder: a systematic review using the appraisal of guidelines for research and evaluation (AGREE II) instrument
Source: Front Psychiatry. 2025 Jun 16;16:1576538. doi: 10.3389/fpsyt.2025.1576538 (PMC12206699; doi:10.3389/fpsyt.2025.1576538)
Supplement: Supplementary file 6 [file DataSheet6.pdf]

Table S7. Interrater reliability using the ICC for AGREE II domain scores

No	Guideline	ICC for AGREE II domain scores						
		Scope and Purpose	Stakeholder Involvement	Rigour of Development	Clarity of Presentation	Applicability	Editorial Independence	Full (95% CI)
1	AAP	−0.333[†]	−3.333[†]	0.818	0.000[¶]	0.289	0.000[‡]	0.750 (0.500–0.886
2	SMOH	0.894	0.874	0.754	0.284	0.375	0.000[§]	0.752 (0.503–0.887)
3	CADDRA	0.290	0.678	0.104	−3.000[†]	0.648	0.000[¶]	0.356 (–0.288–0.706)
4	NICE	0.000[¶]	0.500	0.179	0.000[¶]	0.550	−2.250[†]	0.439 (–0.122–0.744)
5	NHMRC	0.919	0.245	0.376	0.656	−1.350[†]	0.000[¶]	0.390 (–0.221–0.722)
6	MAHTAS	0.000[¶]	0.290	0.728	0.750	0.397	−0.187[†]	0.396 (–0.209–0.724)
7	UMHS	−0.462[†]	0.558	0.280	0.000[¶]	0.321	0.563	0.265 (–0.470–0.665)
8	ICSI	0.711	0.615	0.134	0.000[¶]	0.832	0.667	0.569 (0.138–0.804)
9	IAP	0.677	0.823	0.473	0.346	−0.516[†]	0.562	0.536 (0.072–0.788)
10	BAP	−0.083[†]	0.000[‡]	0.706	−1.105[†]	0.458	−6.000[†]	0.410 (–0.180–0.731)
11	EPA	0.810	0.553	0.852	0.851	0.333	0.000[‡]	0.758 (0.515–0.889)

Note: Full ICC representing overall score from all domains within 23 variable scores among raters
[†]ICC was negative when the within-group variance exceeded the between-group variance
[‡]0 score because of zero agreement among raters
[¶]0 score because of similar scores among raters, resulting lack of variability
[§]0 score because of identical scores among raters, resulting no variability

Abbreviations: ICC, intraclass correlation coefficient; AGREE II, Appraisal of Guidelines for Research and Evaluation Instrument Version II; AAP, American Academy of Pediatrics; SMOH, Singapore Ministry of Health; CADDRA, Canadian ADHD Resource Alliance; NICE, National Institute of Health and Care Excellence; NHMRC, National Health Medical Research Center; MAHTAS, Malaysian Health Technology Assessment Section; UMHS, University of Michigan Health System; ICSI, Institute of Clinical System Improvement; IAP, Indian Academy of Pediatrics; BAP, British Association for Psychopharmacology; EPA, European Psychiatric Association
